# Local energy decomposition analysis of hydrogen-bonded dimers within a domain-based pair natural orbital coupled cluster study

**DOI:** 10.3762/bjoc.14.79

**Published:** 2018-04-25

**Authors:** Ahmet Altun, Frank Neese, Giovanni Bistoni

**Affiliations:** 1 Max Planck Institute for Chemical Energy Conversion, Stifstrasse 34-36, D-45470 Mülheim an der Ruhr, Germany; 2 Max Planck Institute for Coal Research, Kaiser-Wilhelm-Platz 1, D-45470 Mülheim an der Ruhr, Germany

**Keywords:** DLPNO-CCSD(T), hydrogen-bond interaction, interaction energy, local energy decomposition, London dispersion

## Abstract

The local energy decomposition (LED) analysis allows for a decomposition of the accurate domain-based local pair natural orbital CCSD(T) [DLPNO-CCSD(T)] energy into physically meaningful contributions including geometric and electronic preparation, electrostatic interaction, interfragment exchange, dynamic charge polarization, and London dispersion terms. Herein, this technique is employed in the study of hydrogen-bonding interactions in a series of conformers of water and hydrogen fluoride dimers. Initially, DLPNO-CCSD(T) dissociation energies for the most stable conformers are computed and compared with available experimental data. Afterwards, the decay of the LED terms with the intermolecular distance (*r*) is discussed and results are compared with the ones obtained from the popular symmetry adapted perturbation theory (SAPT). It is found that, as expected, electrostatic contributions slowly decay for increasing *r* and dominate the interaction energies in the long range. London dispersion contributions decay as expected, as *r*^−6^. They significantly affect the depths of the potential wells. The interfragment exchange provides a further stabilizing contribution that decays exponentially with the intermolecular distance. This information is used to rationalize the trend of stability of various conformers of the water and hydrogen fluoride dimers.

## Introduction

Hydrogen bonds are of fundamental importance for regulating molecular properties like polarizability [1] and in various biochemical processes, including protein folding [2] and stability [3], replication of DNA and RNA [4], enzyme catalysis [5], proton relay mechanism [6], and drug delivery [7].

Energy decomposition analysis (EDA) schemes have been instrumental in providing insights into the nature of these interactions, by partitioning the total interaction energy of two (or more) interacting fragments into several chemically meaningful contributions [8–10]. EDA methods are mainly based on an early variational study of Morokuma [11]. They are typically carried out at the Hartree–Fock (*HF*) or density functional theory (DFT) level. In these schemes, the interacting system is treated as a supermolecule and the overall interaction energy is decomposed into various terms such as electrostatic interaction, charge transfer, polarization, and the so-called Pauli or exchange-repulsion terms [12–16].

Instead of decomposing DFT or *HF* interaction energies, the widely used symmetry-adapted perturbation theory (SAPT) [17] provides a perturbative expansion of the interaction energy based on the wave functions of the monomers. For weakly interacting monomers, this approach permits to obtain accurate interaction energies as well as their constituting electrostatic, induction, dispersion, and exchange-repulsion terms [9–10].

Although these schemes provide different quantitative estimates for the important components of the interaction, they also provide useful interpretative frameworks in which to discuss experimental observables. For instance, they can be used for discussing trends of dissociation energies [8–9] or the relative stability of conformers [11,15,18–19]. However, two fundamental aspects must be considered when an EDA scheme is applied to a specific chemical problem. The chosen approach must provide: (i) a sufficiently accurate estimate for the observables of interest, which are typically relative energies; (ii) a useful decomposition of the observable into a series of chemically meaningful terms representing the correct physics in the asymptotic region.

In order to address the first issue, the coupled-cluster method with single, double, and perturbative treatment of triple excitations [CCSD(T)] has proven its reliability in a wide range of contexts. This method typically allows for the calculation of relative energies with chemical accuracy (1 kcal/mol) [20–22]. Moreover, our group has recently developed the domain-based local pair natural orbital CCSD(T) method [DLPNO-CCSD(T)] [23–30], which scales linearly with system size and typically provides around 99.9% of the canonical CCSD(T) correlation energy if TightPNO settings are used [31–32]. Thus, DLPNO-CCSD(T) single-point energies can now be obtained for systems with hundreds of atoms and thousands of basis functions while essentially retaining the accuracy and reliability of canonical CCSD(T).

However, the CCSD wave function is a highly complex object that is nonlinear in its parameters (cluster amplitudes). Hence, its direct physical interpretation is not immediately apparent. In order to facilitate the interpretation of DLPNO-CCSD(T) results, we have thus recently introduced the local energy decomposition (LED) analysis scheme, which decomposes the DLPNO-CCSD(T) interaction energy of two or more molecules in terms of electronic and geometric preparation, electrostatic interaction, interfragment exchange, dynamic charge polarization, and London dispersion terms [33].

**Figure 1:**
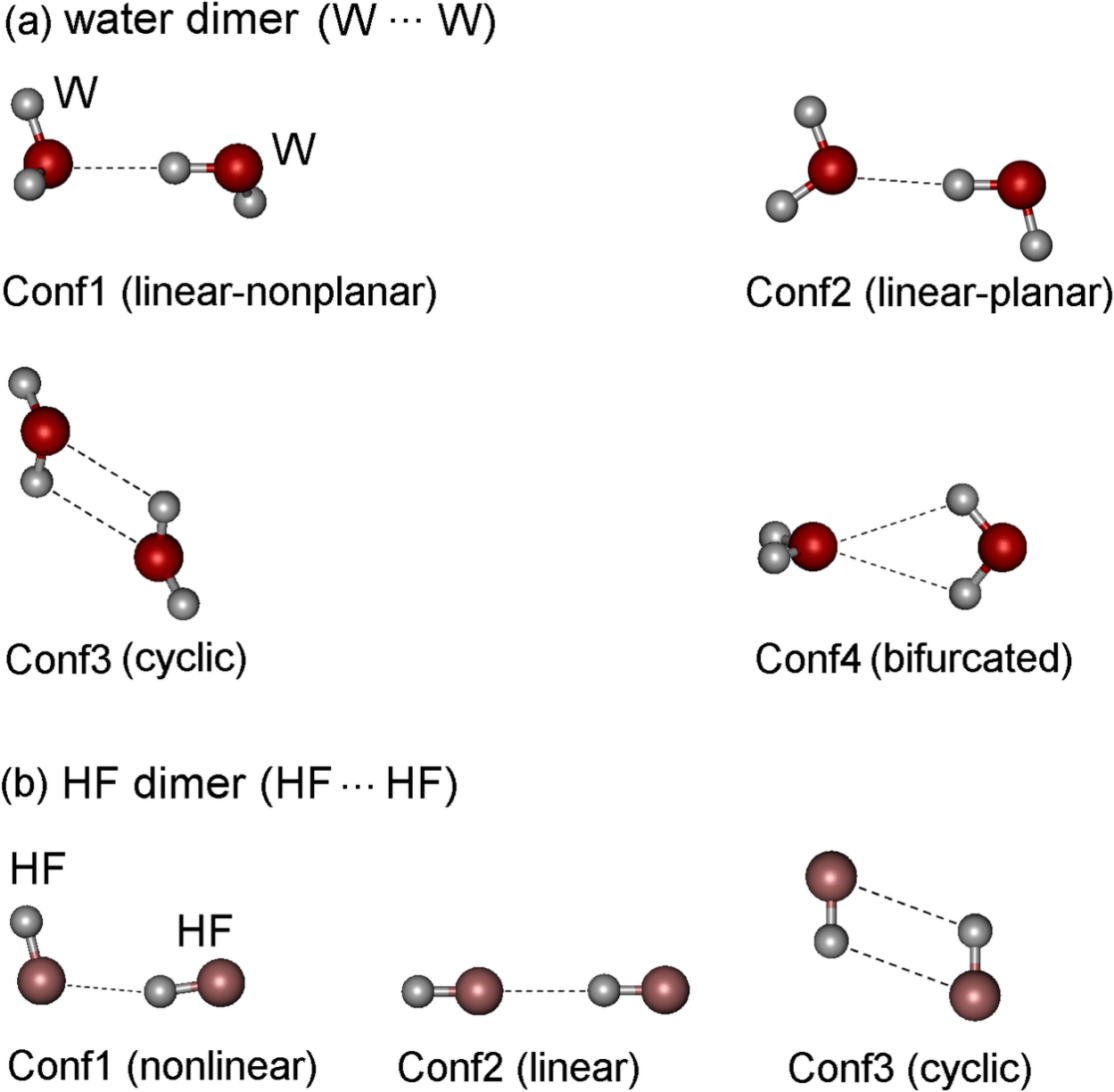
The conformers of (a) water dimer and (b) HF dimer.

Herein, the DLPNO-CCSD(T)/LED methodology is applied to the study of H-bond interactions in a series of conformers of water (H_2_O) and hydrogen fluoride (HF) dimers, which are shown in Figure 1.

These systems are representative examples of H-bond interactions and are often used as model systems for newly developed methods, including EDA schemes [18,34–41]. Although these dimers have been studied extensively, the principal mechanisms of interaction between their constituting monomers are still under debate. The debate concerns the magnitude of individual terms and the importance of London dispersion, charge transfer and polarization effects compared with the dominating electrostatic interaction [15–16,18–19]. Herein, particular emphasis is given in discussing the role played by London dispersion, which constitutes the attractive part of the van der Waals potential and has long been considered a weak effect compared to the other components of the interaction. However, in recent years, several studies have demonstrated that this component of the interaction plays a fundamental role in controlling the stability and reactivity of a wide range of systems [42–43].

This paper is organized as follows. Following a description of the computational details, computed geometries and dissociation energies are compared with previously published experimental and computational data. In the following section, the decay of LED terms with the intermolecular distance between the monomers is discussed for the water dimer case, and results are compared with those obtained from SAPT. This information is then used to rationalize the trends in stability of various conformers of the water and hydrogen fluoride dimers. The last section is devoted to the discussion of the results and concluding remarks.

## Computational Details and Theoretical Aspects

### Computational details

All DLPNO-CCSD(T) calculations and geometry optimizations were performed with a development version of the ORCA 4.0 suite of programs [44–45].

Geometry optimizations and relaxed PES scans constraining only the reaction coordinates were carried out at the RI-MP2 level, employing aug-cc-pVTZ basis set with matching auxiliary basis sets [46–49]. The RIJK approach applying RI approximation for both Coulomb *J* and exchange *K* parts was used [50–51]. Harmonic vibrational frequencies and zero-point energy (ZPE) corrections were computed with the same level of theory used for the geometry optimizations. All valence electrons were included in the correlation treatment (only the core 1s orbitals of oxygen and fluorine atoms were frozen).

Single point DLPNO-CCSD(T) energies and LED calculations employed the Foster–Boys scheme [52] for the localization of the occupied orbitals. All valence electrons were included in the correlation treatment. “TightPNO” settings were used [31–32]. All electron pairs were included in the coupled cluster treatment. The RIJK approximation was used in the *HF* part. The Pipek–Mezey [53] orbital localization scheme was applied for the localization of the PNOs in the LED scheme. In all cases, augmented correlation consistent basis sets of triple-ζ (aug-cc-pVTZ) and quadruple-ζ (aug-cc-pVQZ) qualities were used in conjunction with matching auxiliary basis sets [46–49]. DLPNO-CCSD(T) energies were first corrected for the basis set superposition error (BSSE) [54] and then extrapolated to the complete basis set (CBS) limit using a two-point scheme [55] based on Equation 1 and Equation 2.

[1]
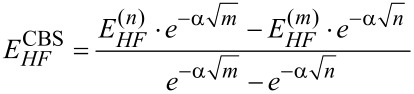


[2]
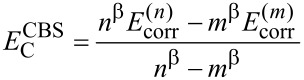


where *E*^(^*^n^*^)^ and *E*^(^*^m^*^)^ are the energies obtained with a basis set of *n*−ζ and *m*−ζ cardinality (here *n* = 3 and *m* = 4), respectively. The previously calibrated values [56] of the constants (α = 5.46, and β = 3.05) for the 3/4 extrapolation were used. The individual LED contributions were also extrapolated.

It is worth mentioning that BSSE-corrected and -uncorrected interaction energies converge to the same value upon extrapolation within 0.1 kcal/mol. For completeness, all energies are reported in Supporting Information File 1.

SAPT calculations were carried out with the MOLPRO [57] program package (version 2012.1) using RI-MP2 geometries. The nondispersive terms of density functional-based SAPT (DFT-SAPT) converge quickly with the basis set size and do not require CBS extrapolation [58–59]. Thus, only the dispersion terms of the DFT-SAPT energies have been extrapolated to CBS limit through Equation 2 using β = 3.05.

DFT-SAPT calculations were carried out with the asymptotically-corrected exchange-correlation functional PBE0AC [60–61]. This functional is a modified PBE0 hybrid functional in which the long-range tail contains 75% of LB94 exchange. The shift parameter applied for the bulk potential within this correction was calculated as the sum of the ionization potential and highest occupied molecular orbital (HOMO) energy of each fragment optimized in the gas phase. The experimentally determined ionization potential of an isolated water molecule was used (0.4638 *E*_h_ [62–63]).

### LED analysis in the DLPNO-CCSD(T) framework

The theory and implementation of the DLPNO-CCSD(T) method and of the LED scheme have been described in detail in a series of recent publications [23–33]. We thus only recall here the main features of this technique.

Within a supramolecular approach, the energy of a molecular adduct *XY* relative to the total energies of noninteracting fragments *X* and *Y*, i.e, dimerization energy (Δ*E*), can be written as:

[3]
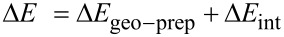


where Δ*E*_geo−prep_ is the geometric preparation energy needed to distort the fragments *X* and *Y* from their structures at infinite separation to their in-adduct geometry. Δ*E*_int_ is the interaction energy of the fragments *X* and *Y* at a given geometry of the adduct *XY*.

Δ*E*_int_ can be decomposed into an *HF* contribution 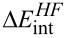 and a correlation contribution 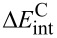:

[4]
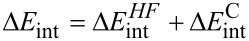


By exploiting the localization of the occupied orbitals in the DLPNO-CCSD(T) framework, the 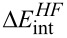 is then decomposed into three contributions [33,64]:

[5]
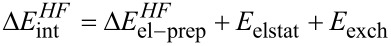


The electronic preparation 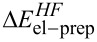 is positive and thus repulsive. It corresponds to the energy needed to bring the electronic structures of the isolated fragments into the one optimal for the interaction. *E*_elstat_ and *E*_exch_ are the electrostatic and exchange interactions, respectively, between the interacting fragments. It is worth noting here that the intermolecular exchange describes a stabilizing component of the interaction, lowering the repulsion between electrons of the same spin.

The DLPNO-CCSD(T) correlation energy (*E*_C_) can be written as a sum of electron-pair correlation energy (ε*_ij_*, where *i* and *j* denote the localized orbitals) contributions plus a perturbative triples correction (*E*_C−(T)_). Local second-order many-body perturbation theory is used to divide the ε*_ij_* terms into “weak pairs”, with expected negligible contribution to the correlation energy, and “strong pairs”. The contribution coming from the weak pairs is kept at the second-order level, whereas the strong pairs are treated at the coupled cluster level. Hence, the overall correlation energy reads [30]:

[6]
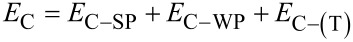


where *E*_C−SP_ and *E*_C−WP_ are the strong-pairs and weak-pairs components of the correlation energy, respectively. The correlation contribution to the interaction energy 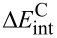 can thus be expressed as a sum of three contributions:

[7]
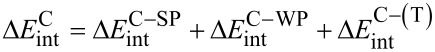


in which 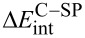, 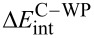 , and 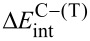 are the strong pairs, weak pairs and triples correction components of the correlation contribution to the interaction energy, respectively.

The 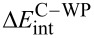 and 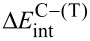 terms can be further divided into electronic preparation and interfragment interaction based on the localization of the occupied orbitals [30]. However, these terms are very small for the systems studied in this work and thus are not decomposed herein.

For the dominant strong pairs contribution 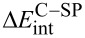, the decomposition exploits the localization of both the occupied and the virtual orbitals in the DLPNO-CCSD(T) framework. Hence, the 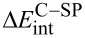 term is divided into three contributions: the electronic preparation energy 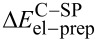, the charge transfer or charge polarization contribution (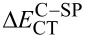), and London dispersion (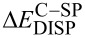).

[8]
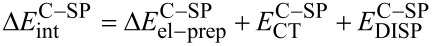


**Figure 2:**
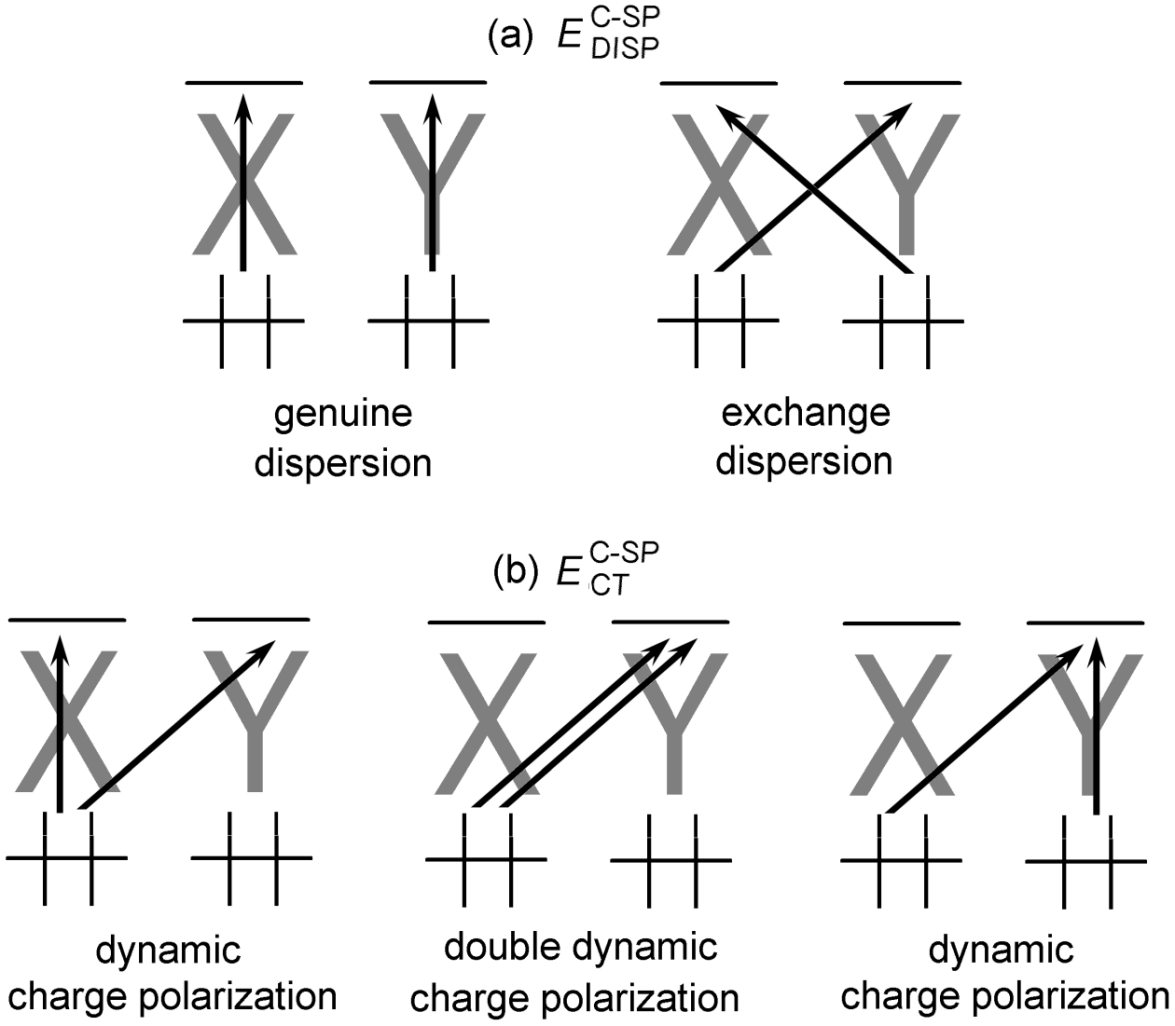
Schematic representation of strong pair excitations in the framework of the DLPNO-CCSD(T) method. Electronic preparation arises from excitations occurring within the same fragment, which are not shown. Only the charge transfer excitations from *X* to *Y* are shown. Analogous charge transfer excitations also exist from *Y* to *X*.

The relevant pair excitation contributions constituting these terms are shown pictorially in Figure 2.

It may be useful to combine several terms depending on the molecular system of interest. For example, 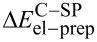 and 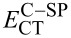 have opposite signs and typically compensate each other [33,64]. Hence, these two terms can be combined to give the SP contribution to the interaction energy excluding dispersion contribution (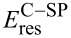):

[9]
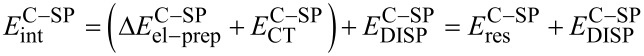


As a final remark, it is worth underscoring that one of the aims of this paper is to discuss the decay of the different components of 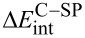 with the distance between the interacting fragments. Hence, we decided to include all electron pairs in the coupled cluster treatment. In this case, the weak-pair contribution only corrects for the pair natural orbital (PNO) truncation and only marginally affects the overall correlation energy. The latter is thus dominated by the strong pairs irrespective of the distance between the fragments.

### SAPT analysis

Symmetry-adapted perturbation theory (SAPT) is a well-established method for the calculation of interaction energies in the context of weak intermolecular interactions [65–66]. It expresses the interaction energy in various terms in the perturbation series that are physically meaningful. In this work, the terms of SAPT are compared with those from our LED scheme.

In SAPT, the non-dispersive interaction energy includes the first order perturbative terms polarization (*E*_pol_) and exchange-repulsion (*E*_exch_), and the second order terms induction (*E*_ind_) and exchange-induction (*E*_exch–ind_). In the DFT variant of SAPT (DFT-SAPT), the difference between the *HF* interaction energy and the sum of the non-dispersive HF-SAPT terms (denoted as δ(*HF*)) is also included in the nondispersive interaction energy to approximately account for the effect of the higher order terms.

[10]
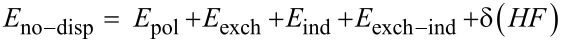


The dispersive energy (*E*_disp_) of the DFT-SAPT includes both the genuine dispersion and its exchange correction, which are calculated both at the second order: the sum of the nondispersive (*E*_no–disp_) and dispersive (*E*_disp_) terms is the DFT-SAPT interaction energy.

[11]
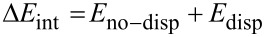


## Results and Discussion

### Geometries and dissociation energies: comparison with experiment

In this section, the computed geometries and dissociation energies for the water and HF dimers in their global minimum (Conf1 of Figure 1) are compared with available experimental data and previously published computational predictions.

For the water dimer in its global minimum, experiments estimate an *r*_0_(O···O) distance between 2.946 and 2.976 Å [67]. The CCSD(T)-based best estimate of *r*_e_(O···O) reported in literature is 2.912 ± 0.005 Å [68]. The *r*_e_(O···O) distance calculated in this work at the RI-MP2/aug-cc-pVTZ level (2.908 Å) is very close to the CCSD(T) prediction. The effect of excitations beyond CCSD(T) has been shown to be negligible by means of CCSDTQ calculations [69].

For the HF dimer, the *r*_e_(F···F) distance of Conf1 calculated at the RI-MP2/aug-cc-pVTZ level (2.746 Å) agrees reasonably well with its previous best estimate (2.735 ± 0.010 Å) on a theoretical potential energy surface refined by comparing multidimensional nuclear quantum energy levels with the corresponding experimental data [70] and the CCSD(T) result of 2.737 Å with a quintuple−ζ basis [71].

The equilibrium Δ*E*_e_ and zero-point corrected Δ*E*_0_ dimerization energies of water and HF dimers are given in Table 1. These correspond to the equilibrium *D*_e_ and zero-point *D*_0_ dissociation energies with opposite sign, respectively.

**Table 1:** The DLPNO-CCSD(T) dimerization energies (kcal/mol) of the conformers of water and HF dimers together with the individual LED terms.

	water dimer					HF dimer		
			
	Conf1	Conf2	Conf3	Conf4		Conf1	Conf2	Conf3

Δ*E*_e_	−4.95	−4.38	−4.15	−3.16		−4.51	−3.56	−3.52
Δ*E*_o_	−2.82*^a^*	−2.86	−2.22	−1.80		−2.69*^b^*	−2.45	−2.07
decomposition of Δ*E*								
Δ*E*_geo–prep_	0.07	0.05	0.05	0.15		0.11	0.04	0.09
Δ*E*_int_	−5.01	−4.43	−4.20	−3.31		−4.62	−3.60	−3.61
decomposition of 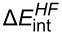								
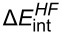	−3.67	−3.30	−2.70	−2.51		−3.89	−3.33	−2.74
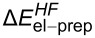	22.91	18.33	16.52	8.74		20.52	10.99	13.94
*E*_elstat_	−22.83	−18.60	−16.43	−9.75		−21.22	−12.56	−14.47
*E*_exch_	−3.76	−3.03	−2.79	−1.50		−3.19	−2.22	−2.22
decomposition of 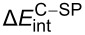								
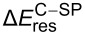	0.19	0.23	0.16	0.26		0.42	0.64	0.31
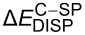	−1.24	−1.10	−1.36	−0.89		−0.94	−0.80	−0.96
WP and triple corrections								
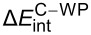	−0.08	−0.08	−0.08	−0.06		−0.08	−0.07	−0.08
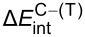	−0.22	−0.17	−0.22	−0.11		−0.13	−0.05	−0.13

^a^Experiment: −3.16 ± 0.03 kcal/mol [72–73]. When the experimentally determined ZPE (1.72 kcal/mol [71]) is used, the resulting value (−3.23 kcal/mol) is very close to the experiment. ^b^When the effect of the anharmonicity of the vibrational energy levels estimated to be −0.185 ± 0.019 kcal/mol [74] is included, the resulting value (−2.88 kcal/mol) agrees reasonably well with the value found on an empirical potential (−3.036 ± 0.003 kcal/mol) [70].

The Δ*E*_e_ value of the water dimer calculated previously at the CCSD(T)/CBS level (−5.01 kcal/mol [68]) agrees remarkably well with the present DLPNO-CCSD(T) result (Δ*E*_e_ = −4.95 kcal/mol, Table 1). The accurate calculation of ZPE correction of H-bonded systems requires larger basis sets and the inclusion of anharmonic effects [68,71]. Thus, the present RI-MP2/aug-cc-pVTZ harmonic ZPE contribution (2.13 kcal/mol) is slightly larger than the experimental value of 1.72 kcal/mol [71]. Using the experimentally determined ZPE contribution for correcting the DLPNO-CCSD(T) Δ*E*_e_ value, one obtains a Δ*E*_0_ value of −3.23 kcal/mol, which is very close to the experimental value of −3.16 ± 0.03 kcal/mol [72–73].

For the HF dimer, the present DLPNO-CCSD(T) values of Δ*E*_e_ (−4.511 kcal/mol) and harmonic Δ*E*_e_ (−2.694 kcal/mol) are consistent with the previously calculated Δ*E*_e_ (−4.580 ± 0.004 kcal/mol) and harmonic Δ*E*_0_ (−2.775 ± 0.024 kcal/mol) values at the CCSD(T)/CBS level [74]. It was shown [74] that the effects of quadruple excitations Q (−0.008 ± 0.004 kcal/mol), relativity (0.016 ± 0.001 kcal/mol), and the diagonal Born–Oppenheimer correction (−0.012 ± 0.000 kcal/mol) to the dimerization energy of the HF dimer are negligible while the anharmonic ZPE contribution (−0.185 ± 0.019 kcal/mol) is significant. Adding these corrections to the calculated Δ*E*_e_ energies, the best fully theoretical estimates of Δ*E*_0_ become −2.964 ± 0.047 and −2.883 kcal/mol at the CCSD(T)/CBS [74] and the present DLPNO-CCSD(T) levels, respectively. These results are similar to that calculated on a potential [70] refined by using experimental data (−3.036 ± 0.003 kcal/mol).

The consistency of the present DLPNO-CCSD(T) and the previous experimental or CCSD(T) dimerization energies of the water and HF dimers indicates that the present computational level can be reliably applied to investigation of dissociation and interaction energies of other H-bonded molecules.

### Decay of LED terms with the intermolecular distance

In this section, the decay of the LED terms with the intermolecular distance (*r*) is discussed for the water dimer. However, the derived conclusions are rather general and thus hold true for the HF dimer as well, as shown in Supporting Information File 1. From now on, we use the term “short-range” to indicate the region where *r*_O---H_ ≤ 3.5 Å*,* and *“*long-range” for the region where *r*_O---H _*>* 3.5 Å.

**Figure 3:**
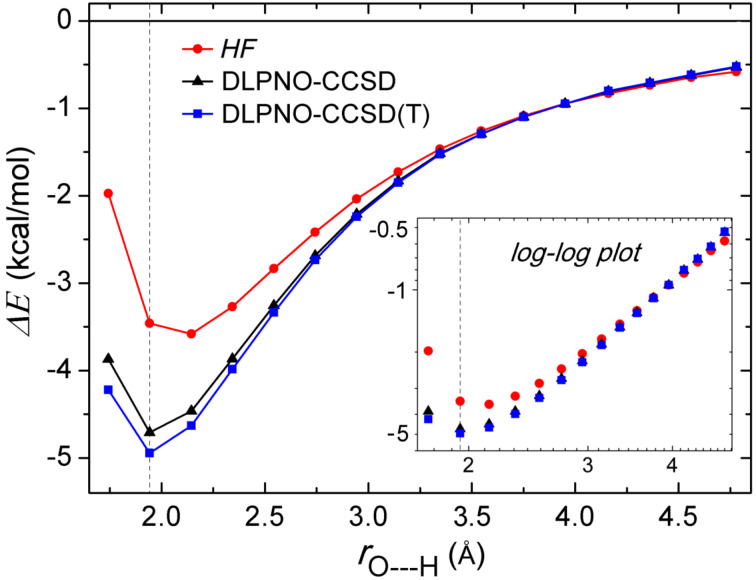
Dissociation curve of Conf1 of water dimer as a function of the H-bond distance. Its nearly linear relation in the log–log scale for the long range is shown as insert on the graph. The black dotted vertical line at 1.943 Å corresponds to the equilibrium *r*_e_(O---H) distance.

Let us start with the analysis of the DLPNO-CCSD(T) energy profile (Figure 3) for the dissociation of the water dimer as a function of the H-bond distance *r*_O---H_ . The corresponding *HF* and DLPNO-CCSD profiles are also reported for comparison.

In the long range, the *HF* and coupled cluster energies show smooth polynomial decays, which are evident from their linear log–log relation shown in the insert of Figure 3. In this range, the correlation contribution to the interaction energy is small and positive and the overall interaction is dominated by the *HF* term. Conversely, in the short range, the correlation energy becomes a significant stabilizing component of the interaction. At the equilibrium position, correlation contributes to the interaction energy of the water dimer by −1.34 kcal/mol. Interestingly, the effect of the perturbative triples (T) is small for all distances.

**Figure 4:**
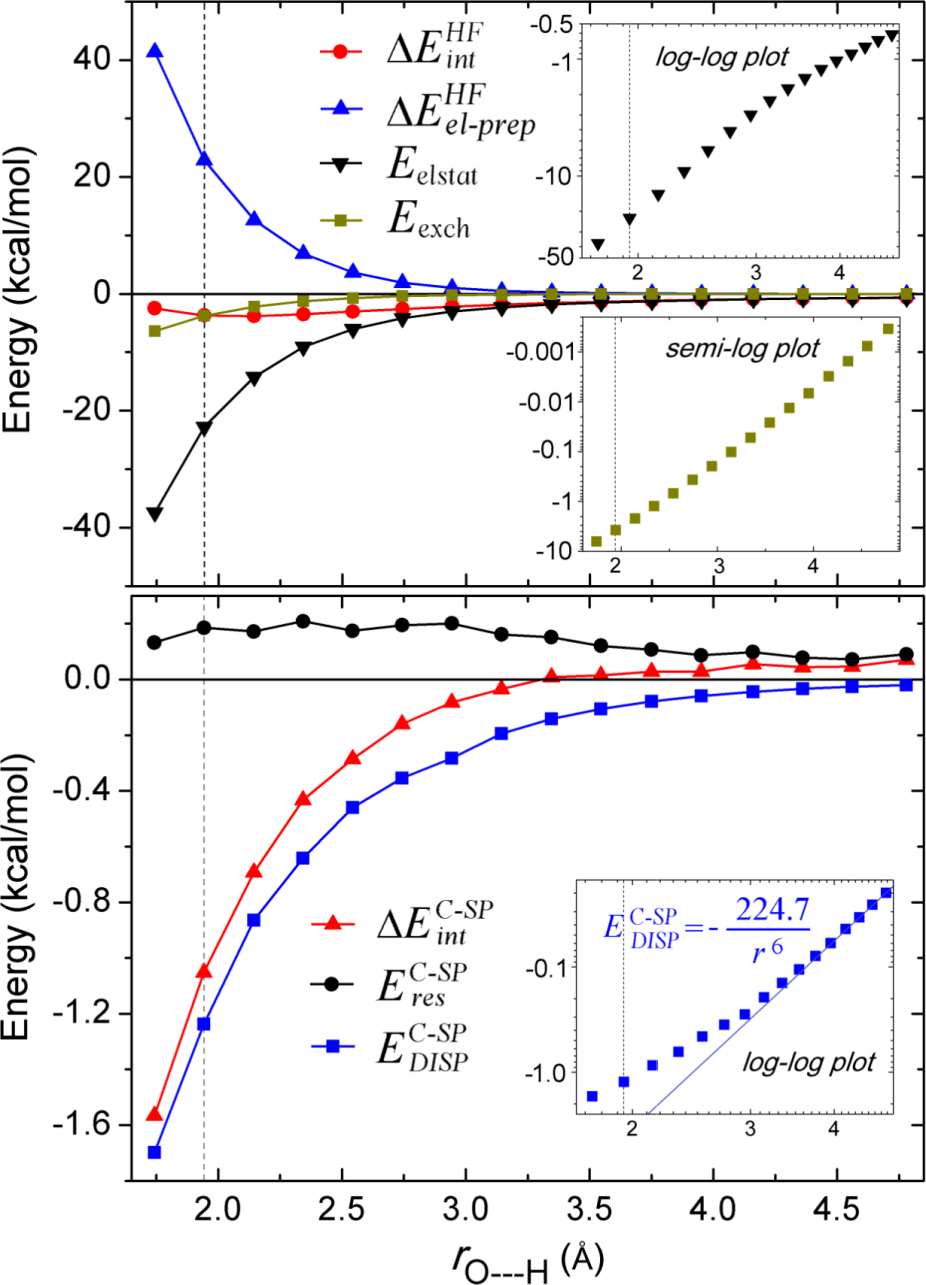
Decomposed *HF* energy terms (top), and correlation energy terms (bottom) of Conf1 of water dimer as a function of the H-bond distance. The nearly linear relation of the long range electrostatic and London dispersion energy terms in the log–log scale as well as the exchange energies in the semi-log scale are given as inserts on the graphs. The black dotted vertical line at 1.943 Å corresponds to the equilibrium *r*_e_(O---H) distance.

A deeper insight into the nature of the water dimer interaction comes by decomposing both the *HF* and correlation component of the dissociation energy into their LED components. Let us start by discussing the *HF* contributions, reported in the upper panel of Figure 4.

In the long range, the only significant LED term is the electrostatic energy, which shows a slow polynomial decay with the distance (indicated by the linear relation in the log–log plot shown in the insert of Figure 4 top). This is not surprising considering the strong dipole of water. However, in the short range, the repulsive electronic preparation arising from distortion of the electronic clouds of the interacting monomers assumes large values and almost entirely counteracts the electrostatic contribution at the equilibrium position. In this position, the remaining *HF* term, i.e., the attractive exchange interaction, amounts to −3.76 kcal/mol, which is very close to the overall *HF* contribution to the interaction energy (−3.67 kcal/mol), and thus provides a fundamental stabilizing component. As expected, the exchange term decays exponentially with intermolecular distance, which is indicated by the linear relation of the semi-log plot shown in the insert of Figure 4 top.

In the correlation part, the weak pair correction is very small in the whole distance range and amounts to −0.08 kcal/mol at the equilibrium position. Hence, only the dominant strong pair contribution is decomposed in the following for the sake of simplicity (lower panel of Figure 4). In the long range, the sum of the dynamic charge polarization and electronic preparation energies (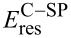) is always positive and provides a small correction to the electrostatics computed at the *HF* level, which is known to overestimate the dipole of water [75]. The remaining correlation term, i.e., London dispersion, is always attractive and decays with *r*^−6^ in the long range, as shown in the log–log insert in the lower panel of Figure 4.

The 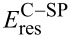 term is dominant in the long range, which makes the overall correlation contribution to the interaction energy positive. However, it reaches just 0.19 kcal/mol at the equilibrium, being much smaller than the corresponding dispersion term (−1.24 kcal/mol). Therefore, the short-range correlation behavior is largely dominated by the London dispersion. The slight fluctuations of the correlation energy terms (Figure 4, bottom panel) arise mainly from difficulties in localizing the PNOs [33].

The *r*^−6^ behavior of the LED estimate of the London dispersion contribution deserves to be discussed in more detail. In order to do that, it is useful to look at the expression for the strong pair correlation energy in the DLPNO-CCSD(T) method [33]:

[12]
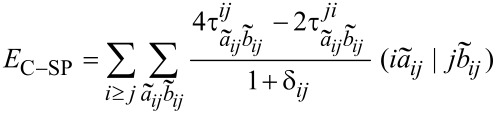


in which 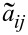 and 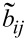
are PNOs that belong to pair *ij*, 
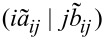
represents the two electron integrals in Mulliken notation, and 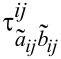 is defined as

[13]
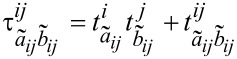


in which 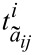
and 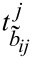
are the singles amplitudes and 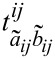 are the corresponding doubles amplitudes. From a multipole expansion of the integrals, it follows that the 
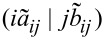
terms decay with 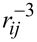, where r*_ij_* is the separation between the charge centroids of the local occupied orbitals 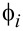 and 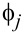. The 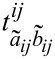 amplitudes also decay with 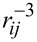, whilst the 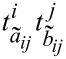 terms have no explicit dependence on the distance between the centroids of 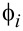 and 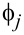. In the LED definition of London dispersion, only the terms of Equation 12, in which 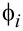 and 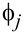 are assigned to different fragments, are included (see Figure 2). Hence, the overall London dispersion consists of two terms, one decaying with *r*^−3^ (due to singles) and the other with *r*^−6^ (due to doubles). Although one could argue that these two terms have different physical meanings, the contribution of the singles to the London dispersion is typically negligible. At the equilibrium distance, it amounts to the 0.34% of the overall London dispersion contribution (see Supporting Information File 1).

### Comparison with DFT-SAPT

**Figure 5:**
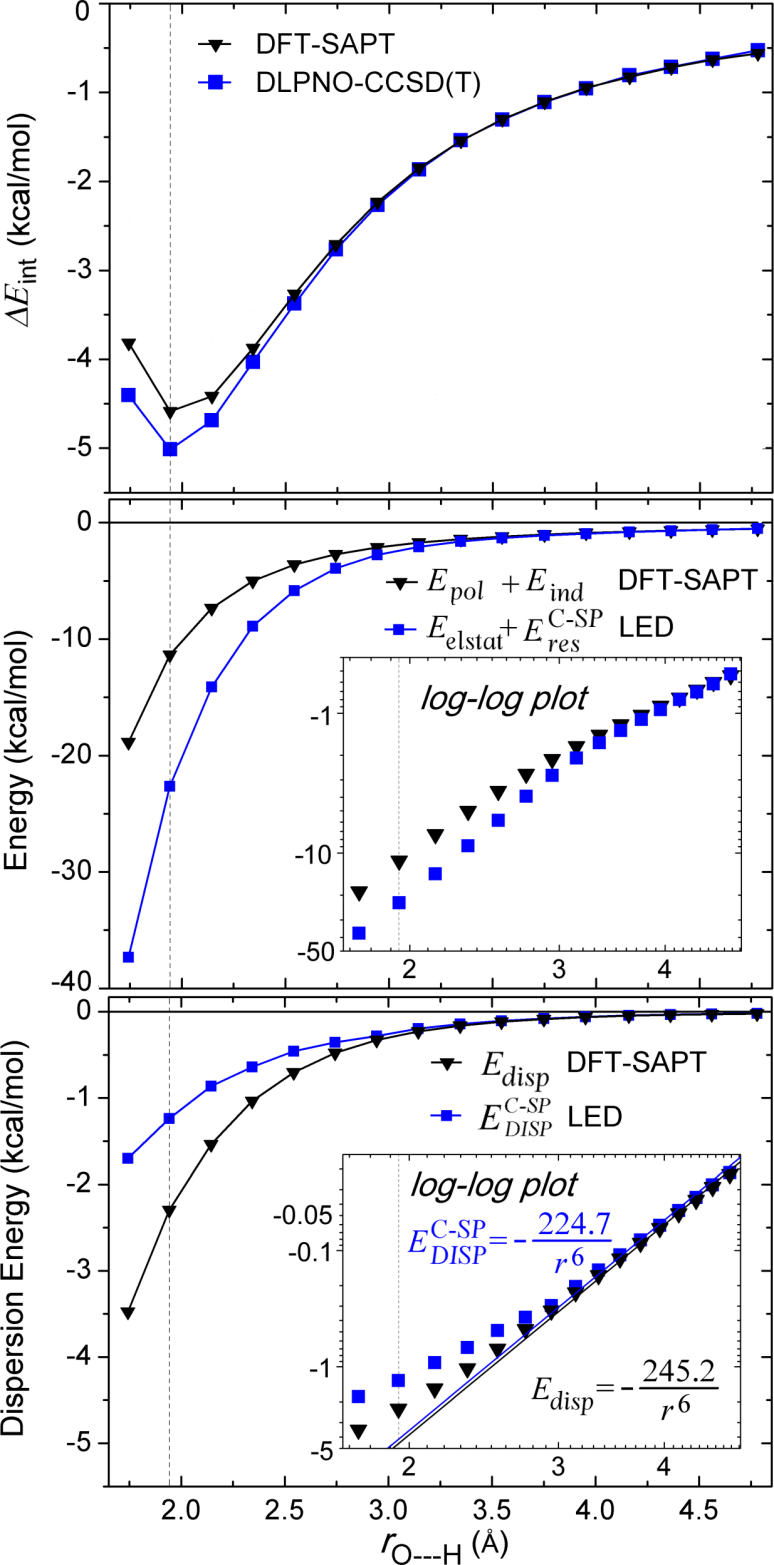
Comparison of total interaction, electrostatic interaction, and London dispersion energies calculated with DLPNO-CCSD(T)/LED and DFT-SAPT for Conf1 of water dimer. The black dotted vertical line at 1.943 Å corresponds to the equilibrium *r*_e_(O---H) distance.

DFT-SAPT treats the interaction energy as a perturbation on the isolated fragments. Hence, the terms of the DFT-SAPT interaction energy are difficult to compare with the ones from energy decomposition schemes based on a supramolecular approach, e.g., the LED. Despite these differences, it is still interesting to compare whether both approaches lead to a similar partitioning of dispersion and electrostatics, in order to draw connections between different interpretative frameworks. A comparison of total interaction, electrostatic, and London dispersion energies calculated with DLPNO-CCSD(T)/LED and DFT-SAPT for the water dimer at various intermolecular distances is reported in Figure 5 (see Supporting Information File 1 for the individual data).

Let us start by discussing the behavior of the total interaction energy (Figure 5 upper panel). At the equilibrium geometry, DFT-SAPT underestimates the interaction energy by 0.42 kcal/mol, whilst the DLPNO-CCSD(T) reproduces the experimental interaction energy within 0.1 kcal/mol (see above). However, the difference between the DFT-SAPT and DLPNO-CCSD(T) total interaction energies decreases with increasing intermolecular distance.

A comparison of electrostatic interactions estimated by LED and DFT-SAPT is shown in the central panel of Figure 5. At the equilibrium geometry, the sum of *E*_elstat_ and 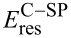 (the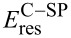 only provides a small correction, see above) is about two times larger than the sum of first-order polarization and second-order induction terms of DFT-SAPT, providing similar results to those recently found using the ALMO-EDA [15] decomposition. Note that in the DFT-SAPT and ALMO-EDA schemes electrostatics and induction are given as separate terms, whilst in LED both effects are included in *E*_elstat_. At least part of the difference between DFT-SAPT and LED/ALMO-EDA arises from the fact that the latter schemes rely on orthogonal orbitals, whilst orbitals belonging to different fragments are not orthogonal in DFT-SAPT. In fact, LED and DFT-SAPT values converge to similar results in the long range, where the overlap between the orbitals is negligible.

Finally, the comparison of London dispersion extracted from LED and DFT-SAPT is shown in the lower panel of Figure 5. At the equilibrium geometry of the water dimer, the DFT-SAPT London dispersion is −1.06 kcal/mol larger than the present DLPNO-CCSD(T)/LED result (Figure 5). It is worth mentioning that the difference in the calculated dispersion energy reduces to about half when a coupled cluster variant of SAPT is used [76]. Again, the difference in the present LED and DFT-SAPT dispersion energies diminishes as the fragments move apart. In the long range, both definitions of dispersion decay with *r*^–6^ dependence with C_6_ coefficients differing only by 8.4%.

These results demonstrate that, despite the non-uniqueness of the definition of the interaction energy terms, both schemes represent the correct physics in the asymptotic region.

### Relative stabilities of the conformers

The four conformers of water dimer (Conf1: nonplanar with a nearly linear OH···O bond; Conf2: planar-Conf1; Conf3: cyclic; and Conf4: bifurcated) and the three conformers of HF dimer (Conf1: nonlinear; Conf2: linear; Conf3: cyclic) investigated are shown in Figure 1. In all cases, Conf1 corresponds to the absolute minimum whilst the other conformers are not stable intermediates and present at least one imaginary frequency.

The dimerization energies and their constituting LED contributions for the conformers of water and HF dimers are shown in Table 1. In all cases, the dimerization energies of the conformers are quite similar. For the water conformers, they range from −4.95 kcal/mol (Conf1) to −3.16 kcal/mol (Conf4). The situation is similar for the HF conformers, for which the dimerization energies range from −4.51 kcal/mol (Conf1) to −3.52 kcal/mol (Conf3). Therefore, the conformers of both dimers lie within 2 kcal/mol. The inclusion of the ZPE correction to relative energies, which amounts up to 0.7 kcal/mol, makes the energetic separation between the conformers even smaller.

These results already suggest that subtle differences in the various terms of the interaction determine the trend in the interaction energies. Deeper insight into this aspect can be obtained by looking at the individual contributions from the LED decomposition. Consistent with what was discussed in the previous sections, the geometric preparation, weak pairs, and perturbative triples do not contribute significantly to the relative stabilities of the different conformers. In all cases, the largest LED terms are electronic preparation and electrostatic interactions at the *HF* level. This is not surprising, considering the strong dipole moments of water and HF and in light of the fact that the electrostatic interaction is well described at the HF level, as shown in the previous section. Interestingly, *E*_elstat_ and Δ*E*_e_ show similar trends, thus highlighting the importance of classical electrostatic interactions in determining the relative stabilities of different conformers. However, as *E*_elstat_ and 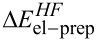 largely cancel each other, the other contributions of the interaction also play an important role. In particular, the interfragment exchange energy provides a fundamental stabilizing component for all conformers and is typically of the same order of the overall 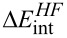.

Electron correlation also affects the energetic separation of various conformers. For example *HF* predicts a large energetic separation between Conf2 and Conf3 for both water and HF dimers (about 0.6 kcal/mol) whilst the inclusion of electron correlation makes them virtually degenerate. The LED decomposition of the strong pairs shows that 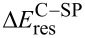, i.e., the sum of the counteracting dynamic electronic preparation and dynamic charge polarization, is positive and ranges from 0.16 to 0.64 kcal/mol. Hence, the major correlation contribution to Δ*E*_e_ in all cases arises from the London dispersion 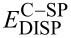, which ranges from −0.9 to 1.4 kcal/mol. However, it is worth underscoring that London dispersion and 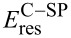 show similar variations among the various conformers. This picture holds true for all conformers of water and HF dimers. In brief, the LED analyses show that the energetic ordering for the conformers of the water and HF dimers arises from a balance of the stabilizing electrostatic (which is dominated by the *HF* contribution), interfragment exchange, and dispersion terms, which are partially counteracted by the positive electronic preparation.

## Conclusion

The recently developed LED scheme in the DLPNO-CCSD(T) framework is a useful and affordable tool to accurately quantify interaction energies and provides their decomposition into physically meaningful terms. In this work, this scheme was applied to the study of H-bond interactions on a series of prototype molecular systems, i.e., a series of conformers of water and HF dimers. For the water dimer, results are compared to the ones obtained from the popular DFT-SAPT approach.

The dissociation energy of water and HF dimers in their equilibrium structure was computed at the DLPNO-CCSD(T) level and results were found to be in perfect agreement with available experimental and previously available CCSD(T) data. On the other hand, the DFT-SAPT was found to underestimate the interaction energy in the water dimer by 0.42 kcal/mol.

For the water dimer, the decay of the different LED components with the intermolecular distance was studied. It was found that, when the water dimer is in its equilibrium structure, the electrostatic interaction estimated via the LED scheme is about twice as large as that obtained from DFT-SAPT. This difference mainly arises from the fact that LED uses orthogonal orbitals whilst the orbitals of different fragments are non-orthogonal in DFT-SAPT. However, both schemes converge to the same asymptotic value. The London dispersion interaction calculated by DFT-SAPT and LED schemes differ by 1 kcal/mol in the equilibrium position, but also converge to the same values in the long range, showing in both cases the expected *r*^−6^ decay. The LED analysis demonstrates the presence of another stabilizing contribution in the short range, i.e., the interfragment exchange. This component of the interaction decays exponentially and acts by lowering the repulsion of electrons with the same spin.

In the last part of the paper, the DLPNO-CCSD(T)/LED scheme is used to rationalize the trend of stability of a series of conformers of water and HF dimers. It was found that the energetic separation introduced by different H-bond networks arise from a balance between many terms.

## Supporting Information

File 1 The Cartesian coordinates of the optimized structures; the individual and total DLPNO-CCSD(T)/LED energies computed with aug-cc-pVTZ and aug-cc-pVQZ; and HF-SAPT and DFT-SAPT energies computed with aug-cc-pVTZ and aug-cc-pVQZ. The energetics includes CBS, BSSE, and BSSE-followed CBS corrected values.
Additional data.
