# Supplementary material for: Local energy decomposition analysis of hydrogen-bonded dimers within a domain-based pair natural orbital coupled cluster study
Source: Beilstein J Org Chem. 2018 Apr 25;14:919–29. doi: 10.3762/bjoc.14.79 (PMC5942370; doi:10.3762/bjoc.14.79)
Supplement: File 1 — The Cartesian coordinates of the optimized structures; the individual and total DLPNO-CCSD(T)/LED energies computed with aug-cc-pVTZ and aug-cc-pVQZ; and HF-SAPT and DFT-SAPT energies computed with aug-cc-pVTZ and aug-cc-pVQZ. The energetics includes CBS, BSSE, and BSSE-followed CBS corrected values. Additional data. [file Beilstein_J_Org_Chem-14-919-s001.pdf]

Supporting Information

for

Local energy decomposition analysis of hydrogen-bonded dimers within a domain-based pair natural orbital coupled cluster study

Ahmet Altun[1,2], Frank Neese[*1,2] and Giovanni Bistoni[*1,2]

Address: [1]Max Planck Institute for Chemical Energy Conversion, Stifstrasse 34-36, D-45470 Mülheim an der Ruhr, Germany and [2]Max Planck Institute for Coal Research, Kaiser-Wilhelm-Platz 1, D-45470 Mülheim an der Ruhr, Germany

Email: Frank Neese* - Frank.Neese@kofo.mpg.de; Giovanni Bistoni* - giovanni.bistoni@kofo.mpg.de

* Corresponding author

Additional data

Contents

1. RI-MP2/aug-cc-pVTZ Cartesian Coordinates in Å
    1.1. Relaxed PES Scan on Conf1 (s0) of W---W
    1.2. Higher Energy Conformers of W---W
    1.3. Relaxed PES Scan on Conf1 (s0) of HF---HF
    1.4. Higher Energy Conformers of HF---HF
    1.5. Isolated H_2O and HF

2. DLPNO-CCSD(T)/LED Energies of W---W (kcal/mol)
    2.1. aug-cc-pVTZ Energies
    2.2. aug-cc-pVQZ Energies
    2.3. CBS-Only Corrected Energies
    2.4. BSSE-Corrected aug-cc-pVTZ Energies
    2.5. BSSE-Corrected aug-cc-pVQZ Energies
    2.6. BSSE-Followed CBS Energies

3. SAPT Energies of W---W (kcal/mol)
    3.1. HF-SAPT Energies
    3.2. DFT-SAPT/aug-cc-pVTZ Energies
    3.3. DFT-SAPT/aug-cc-pVQZ and DFT-SAPT/CBS Energies

4. DLPNO-CCSD(T)/LED Energies of HF---HF (kcal/mol)
    4.1. aug-cc-pVTZ Energies
    4.2. aug-cc-pVQZ Energies
    4.3. CBS-Only Corrected Energies
    4.4. BSSE-Corrected aug-cc-pVTZ Energies
    4.5. BSSE-Corrected aug-cc-pVQZ Energies
    4.6. BSSE-Followed CBS Energies
    4.7. Plot of LED Terms

5. The Effect of Augmented Functions and Correction Scheme on ΔE_{int}

1. RI-MP2/aug-cc-pVTZ Cartesian Coordinates in Å

1.1. Relaxed PES Scan on Conf1 (s0) of W---W

**s-1**	r(O---O)=2.914 Å	r(O---H)=1.743 Å	θ(O-H---O)=173.9°
O	-0.03241427916612	-0.03923306664757	0.09464467811048
H	0.92911308755271	-0.04210067555493	0.05351171314230
H	-0.27796349719828	0.88960788085644	0.03863077635921
O	-0.94672646959820	-1.24316021857156	2.34907943368972
H	-0.65655406804927	-0.85276767354004	1.50413398084353
H	-1.48916034432929	-1.99504102528870	2.09946196892150
**s0**	r(O---O)=2.908 Å	r(O---H)=1.943 Å	θ(O-H---O)=173.6°
O	0.00734121594622	0.01158551260826	0.01667101625365
H	0.96920243723402	0.01150817186684	-0.00782003942165
H	-0.23653892850061	0.94167735415384	-0.01792651828719
O	-0.98963357612938	-1.29467086163939	2.41546057115780
H	-0.69832831461969	-0.90500029812740	1.57798060549298
H	-1.52574840471901	-2.04779465760851	2.15509691587116
**s1**	r(O---O)=3.108 Å	r(O---H)=2.144 Å	θ(O-H---O)=173.9°
O	0.04539786424111	0.05988433281192	-0.07270035519077
H	1.00741367128156	0.06473509993896	-0.05835363257205
H	-0.19872975459475	0.99050512492337	-0.06889725915261
O	-1.02744917467306	-1.34860559819743	2.48167223537494
H	-0.73400227921988	-0.95335809776645	1.64841916112082
H	-1.56633589782342	-2.09585564045673	2.20932240148641
**s2**	r(O---O)=3.308 Å	r(O---H)=2.343 Å	θ(O-H---O)=175.3°
O	0.08559728476288	0.11205417865241	-0.14537160910866
H	1.04736477086297	0.11862747904008	-0.12441418846540
H	-0.15885962290569	1.04241981242603	-0.13465922634379
O	-1.07316458385172	-1.40886632140950	2.55406087954426
H	-0.76677742683774	-0.99761928243620	1.73437773215565
H	-1.60786599281915	-2.14931064501917	2.25546896328468
**s3**	r(O---O)=3.508 Å	r(O---H)=2.543 Å	θ(O-H---O)=176.9°
O	0.12707784092643	0.16564414282623	-0.21240601706901
H	1.08878718209226	0.17401395207820	-0.19637766527853
H	-0.11811225449003	1.09571408119916	-0.20532640580930
O	-1.12118416325739	-1.47140091147992	2.62801463263527
H	-0.80012698133168	-1.04241382007971	1.82425052028723
H	-1.65014719472804	-2.20425222329032	2.30130748630108
**s4**	r(O---O)=3.708 Å	r(O---H)=2.743 Å	θ(O-H---O)= 178.1°
O	0.17173013759813	0.22320580559625	-0.26946102229516
H	1.13338783097275	0.23351548706915	-0.27868180230039
H	-0.07458112740708	1.15273779372572	-0.28593825477016
O	-1.17185690113384	-1.53763618753931	2.70433677287992
H	-0.83697854777899	-1.09170677878146	1.91663554259911
H	-1.69540696303943	-2.26281089881671	2.35257131495343
**s5**	r(O---O)=3.908 Å	r(O---H)=2.944 Å	θ(O-H---O)= 179.6°
O	0.21433332183672	0.27875207264451	-0.33112031739582
H	1.17565602789295	0.28964594395985	-0.35477963649271
H	-0.03279146767264	1.20762061099500	-0.36192738219319
O	-1.22112908125085	-1.60264915498822	2.77890236810689
H	-0.87165781522300	-1.13777198771842	2.00955991148610
H	-1.73811655637163	-2.31829226363907	2.39882760755547
**s6**	r(O---O)=4.108 Å	r(O---H)=3.144 Å	θ(O-H---O)= 179.5°
O	0.26074368730943	0.33950344616319	-0.38558354185515
H	1.22084125662306	0.34958030252568	-0.43841001834393
H	0.01193386170107	1.26641438522760	-0.44564434915550
O	-1.27089499514202	-1.66859679573686	2.85436713260722
H	-0.91223426273317	-1.19183016640581	2.09737956583042
H	-1.78409511854682	-2.37776595052015	2.45735376198369

**s7**	r(O---O)=4.308 Å	r(O---H)=3.346 Å	θ(O-H---O)= 177.1°
O	0.30312706234927	0.39482381069610	-0.44360798642697
H	1.26191024585858	0.40392371721350	-0.51604279105932
H	0.05241428502055	1.31968116075199	-0.52345579648677
O	-1.32198787606083	-1.73635313126089	2.92921727684073
H	-0.94434127850139	-1.23410336917858	2.19899834303622
H	-1.82482800945464	-2.43066696696848	2.49435350516287
**s8**	r(O---O)=4.508 Å	r(O---H)=3.546 Å	θ(O-H---O)= 176.7°
O	0.35230485922621	0.45947459562334	-0.49212158442058
H	1.30726283925144	0.46390483377624	-0.60410296627444
H	0.09742196759875	1.37883788971362	-0.61174322849859
O	-1.37219179399057	-1.80282395957716	3.00504591685669
H	-0.98715670476172	-1.29102599020823	2.28590030445950
H	-1.87134673811256	-2.49106214807417	2.55648410894416
**s9**	r(O---O)=4.708 Å	r(O---H)=3.750 Å	θ(O-H---O)= 173.4°
O	0.39437086383756	0.51439836729412	-0.54838645813315
H	1.34659799662697	0.51585180703046	-0.68149461672085
H	0.13613338080117	1.42976781464193	-0.68934016368712
O	-1.42495156246979	-1.87285194404806	3.07878404621313
H	-1.01706768354299	-1.33005747255141	2.39628903806370
H	-1.90878856604138	-2.53980335111339	2.58361070533104
**s10**	r(O---O)=4.908 Å	r(O---H)=3.951 Å	θ(O-H---O)= 173.4°
O	0.43753938057499	0.57099206050616	-0.61873252060197
H	1.38988808891467	0.57316975090678	-0.75079032745834
H	0.17909131820687	1.48636327257649	-0.75913524075094
O	-1.46884164772777	-1.93139228150847	3.14852749222649
H	-1.05906806102679	-1.38589075411292	2.46982814371777
H	-1.95231464973042	-2.59593682711438	2.64976500393374
**s11**	r(O---O)=5.108 Å	r(O---H)=4.160 Å	θ(O-H---O)= 168.9°
O	0.49212734110347	0.64508620041134	-0.63453976131149
H	1.42697436212740	0.62175353647754	-0.85794888168270
H	0.21805620793805	1.53688600218139	-0.86686107114345
O	-1.53467406037939	-2.01302697799935	3.22786549947062
H	-1.08846883718414	-1.42790514076898	2.60778265672222
H	-1.98772058439383	-2.64548839904830	2.66316410901155
**s12**	r(O---O)=5.308 Å	r(O---H)=4.361 Å	θ(O-H---O)=169.0°
O	0.53546046239191	0.70135886129342	-0.70498551642417
H	1.47073816968568	0.67969117488537	-0.92670270352736
H	0.26011261409318	1.59282769118875	-0.93701949428269
O	-1.57729394658381	-2.07187615626270	3.29755122309392
H	-1.13042787763417	-1.48428294878439	2.68058982760556
H	-2.03229499274123	-2.70041340106682	2.73002921460149
**s13**	r(O---O)=5.508 Å	r(O---H)=4.561 Å	θ(O-H---O)= 169.0°
O	0.57838568084531	0.75771613018541	-0.77560978080308
H	1.51401961317849	0.73683764592391	-0.99585920579879
H	0.30309300132899	1.64950046980868	-1.00645880517727
O	-1.62084160261393	-2.12981515040430	3.36729095234644
H	-1.17294988005733	-1.54049382257830	2.75279829819469
H	-2.07541238346999	-2.75644005168175	2.79730109230477
**s14**	r(O---O)=5.708 Å	r(O---H)=4.779 Å	θ(O-H---O)= 163.4°
O	0.63557104956736	0.83178922190674	-0.79458610118292
H	1.54704810113773	0.78092791000017	-1.09620676901366
H	0.33356255827758	1.69003046140085	-1.10533599847217
O	-1.68286725490918	-2.21480022239309	3.43913573327694
H	-1.20195233399755	-1.57934966335266	2.90041417064045
H	-2.10506769086439	-2.79129248630837	2.79604151581811

1.2. Higher Energy Conformers of W---W

**Conf2**			
O	0.00274693820973	-0.12560418599634	-0.08158540851828
H	-0.00218783072902	0.46124607846354	-0.84269453758102
H	-0.00022083168550	-1.01320061643543	-0.45133671921653
O	-0.00234406894628	-0.08392322392063	2.84299909025522
H	0.00072818153414	0.03617728574566	1.88332399698529
H	0.00127761161693	0.80881266214319	3.19672757807529
**Conf3**			
O	-0.01686802424253	-1.42739417260086	-0.11921231068698
H	-0.57169417383747	-0.78171244983461	0.33466771468510
H	0.30996301584492	-1.99993335952454	0.58043708460294
O	0.01686802424443	1.42739417260388	0.11921231068483
H	0.57169417382952	0.78171244982818	-0.33466771468202
H	-0.30996301583887	1.99993335952796	-0.58043708460387
**Conf4**			
O	-0.00297570560363	-0.00000016482432	-0.03321456607524
H	0.66690441293751	-0.00000011678637	-0.72339175047427
H	-0.83776842935825	0.00000028728913	-0.51114163245838
O	-0.02753537472216	0.00000001141765	2.98195918647067
H	0.02086776160809	-0.74452112926726	2.37429042337839
H	0.02086733513845	0.74452111217117	2.37429033915881

1.3. Relaxed PES Scan on Conf1 (s0) of HF---HF

**s-1**	r(F---F)=2.558 Å	r(F---H)=1.627 Å	θ(F-H---F)=174.2°
H	-0.30922688799115	0.84097522313390	-0.00000458848094
F	0.06205686365493	-0.00722720612764	0.00000174328127
H	1.68889641283860	-0.03007883240806	-0.00000001352104
F	2.61919661149762	0.05172481540182	0.00000285872070
**s0**	r(F---F)=2.746 Å	r(F---H)=1.827 Å	θ(F-H---F)=170.4°
H	-0.38796278517536	0.86330637901020	-0.00000977491860
F	-0.04061354010285	0.00605168994919	0.00003560268516
H	1.78453684015533	-0.06619212709085	-0.00007106862474
F	2.70496248512288	0.05222805813148	0.00004524085818
**s1**	r(F---F)=2.946 Å	r(F---H)=2.026 Å	θ(F-H---F)=171.2°
H	-0.50646331964227	0.87677299821240	-0.00000062935907
F	-0.13403876507638	0.03093050659477	0.00000673709093
H	1.88947982288721	-0.07353890399877	-0.00000507109517
F	2.81194526183144	0.02122939919162	-0.00000103663669
**s2**	r(F---F)=3.146 Å	r(F---H)=2.227 Å	θ(F-H---F)=171.6°
H	-0.62852103592066	0.88975262357358	0.00000027274163
F	-0.22592260495660	0.05846383944582	0.00000489411099
H	1.99600248639148	-0.08429966405052	-0.00000077970662
F	2.91936415448578	-0.00852279896886	-0.00000438714600
**s3**	r(F---F)=3.346 Å	r(F---H)=2.427 Å	θ(F-H---F)=171.9°
H	-0.75421129207609	0.90156970932535	0.00000056789741
F	-0.31610051833418	0.08890166078145	0.00000498132861
H	2.10374351689593	-0.09705471828683	-0.00000108618773
F	3.02749129351434	-0.03802265181996	-0.00000446303829
**s4**	r(F---F)=3.546 Å	r(F---H)=2.627 Å	θ(F-H---F)=172.2°
H	-0.88250531522446	0.91182015347593	0.00000088762176
F	-0.40483847291198	0.12210327404342	0.00000502784930
H	2.21218367986377	-0.11092146708175	-0.00000134610274
F	3.13608310827266	-0.06760796043757	-0.00000456936831

**s5**	r(F---F)=3.746 Å	r(F---H)=2.828 Å	θ(F-H---F)=172.5°
H	-1.01404754252073	0.91968077028876	0.00000123813114
F	-0.49182675892965	0.15895944966864	0.00000503203724
H	2.32144274319955	-0.12531695507441	-0.00000157753718
F	3.24535455825083	-0.09792926488297	-0.00000469263120
**s6**	r(F---F)=3.946 Å	r(F---H)=3.028 Å	θ(F-H---F)=172.9°
H	-1.14945062505643	0.92403219831924	0.00000162135980
F	-0.57672545771356	0.20078348110411	0.00000500566998
H	2.43166368999229	-0.14001275121138	-0.00000183757266
F	3.35543539277769	-0.12940892821195	-0.00000478945711
**s7**	r(F---F)=4.146 Å	r(F---H)=3.227 Å	θ(F-H---F)=173.5°
H	-1.29088662766482	0.92218245551022	0.00000207167573
F	-0.65840136849053	0.25074996109589	0.00000490086148
H	2.54341133268920	-0.15352044208303	-0.00000208568524
F	3.46679966346615	-0.16401797452306	-0.00000488685197
**s8**	r(F---F)=4.346 Å	r(F---H)=3.430 Å	θ(F-H---F)=172.1°
H	-1.40129748537154	0.93550378032620	0.00000231199713
F	-0.75269788375276	0.27969611579559	0.00000499972520
H	2.64582341146548	-0.18143799355162	-0.00000223920302
F	3.56909495765881	-0.17836790257015	-0.00000507251932
**s9**	r(F---F)=4.546 Å	r(F---H)=3.623 Å	θ(F-H---F)=179.0°
H	-1.65518292183663	0.78374915348093	0.00000418669249
F	-0.77311633318389	0.51458196198963	0.00000336996561
H	2.78942051133782	-0.14565710984949	-0.00000289808481
F	3.69980174368269	-0.29728000562105	-0.00000465857329
**s10**	r(F---F)=4.746 Å	r(F---H)=3.823 Å	θ(F-H---F)=179.3°
H	-1.75324748013438	0.80294296986098	0.00000435290980
F	-0.87143938529971	0.53314926139184	0.00000354138074
H	2.88810596307363	-0.16209367342481	-0.00000308891683
F	3.79750390236046	-0.31860455782798	-0.00000480537371
**s11**	r(F---F)=4.946 Å	r(F---H)=4.023 Å	θ(F-H---F)=179.5°
H	-1.85134705355090	0.82214516558830	0.00000451911346
F	-0.96973540326312	0.55181696826994	0.00000371194117
H	2.98671618504409	-0.17906533921849	-0.00000327503629
F	3.89528927176992	-0.33950279463973	-0.00000495601834
**s12**	r(F---F)=5.146 Å	r(F---H)=4.223 Å	θ(F-H---F)=179.8°
H	-1.94946281162390	0.84132532872826	0.00000468547275
F	-1.06800041380492	0.57059125030458	0.00000388160725
H	3.08523999993410	-0.19646313128146	-0.00000345731621
F	3.99314622549471	-0.36005944775136	-0.00000510976379
**s13**	r(F---F)=5.346 Å	r(F---H)=4.424 Å	θ(F-H---F)=179.9°
H	-2.04759113623921	0.86048914395238	0.00000485194735
F	-1.16624434986629	0.58944757388568	0.00000405059371
H	3.18370007506446	-0.21420491903754	-0.00000363651827
F	4.09105841104102	-0.38033779880050	-0.00000526602279
**s14**	r(F---F)=5.546 Å	r(F---H)=4.624 Å	θ(F-H---F)=180.0°
H	-2.14572790914684	0.87964036211225	0.00000501850667
F	-1.26447111189059	0.60836676998114	0.00000421905617
H	3.28210573475277	-0.23222264419655	-0.00000381324171
F	4.18901628628465	-0.40039048789682	-0.00000542432113
**s15**	r(F---F)=5.746 Å	r(F---H)=4.824 Å	θ(F-H---F)=179.9°
H	-2.24387063343383	0.89878186237352	0.00000518512942
F	-1.36268418751715	0.62733432031079	0.00000438711419
H	3.38046634335231	-0.25046167732631	-0.00000398797348
F	4.28701147759867	-0.42026050535797	-0.00000558427013

1.4. Higher Energy Conformers of HF---HF

**Conf2**			
H	-1.01107379043956	-0.00000002158199	0.00000001052485
F	-0.08799911059979	0.00000002098704	-0.00000002295031
H	1.82706278319895	0.00000002270876	0.00000001461498
F	2.75201011784042	-0.00000002211382	-0.00000000218951
**Conf3**			
H	1.00268965422152	0.41260814802418	0.00000615157015
F	1.88614457314300	0.13546434067777	-0.00000615157071
H	1.79731030186484	2.38739171120797	0.00000615156678
F	0.91385547077064	2.66453580009008	-0.00000615156623

1.5. Isolated H_2O and HF

H_2O			
O	0.00613212494937	0.01000989615158	0.01677093730520
H	0.96718697962795	0.01431438113483	-0.00789183784798
H	-0.23331437989769	0.94044676134254	-0.01795464091242

HF			
H	-0.38738075058407	0.86186992308103	-0.00000969888177
F	-0.04119557469414	0.00748814587836	0.00003552664833

2. DLPNO-CCSD(T)/LED Energies of W---W (kcal/mol)

The LED terms of the fragments with H-bond acceptor (always at the left in Figure 1) and donor(always at the right in Figure 1) of the dimers are distinguished to each other by labeling them with *X* and *Y*, respectively.

2.1. aug-cc-pVTZ Energies

$r_{O\cdots H}$	ΔE_{HF}	ΔE_{CCSD}	$\Delta E_{CCSD(T)}$	$\Delta E_{geo-prep}$ HF	$\Delta E_{geo-prep}$ CCSD	$\Delta E_{geo-prep}$ CCSD(T)	ΔE_{int}	ΔE_{int}^{HF}	ΔE_{int}^{C}	E_{elstat}	E_{exch}
PES	on Conf1										
1.743	-2.057	-4.041	-4.389	0.426	0.165	0.128	-4.517	-2.483	-2.034	-37.395	-6.389
1.943	-3.500	-4.843	-5.084	0.194	0.056	0.036	-5.120	-3.695	-1.425	-22.743	-3.763
2.144	-3.626	-4.620	-4.795	0.195	0.046	0.026	-4.821	-3.821	-1.000	-14.217	-2.196
2.343	-3.325	-4.041	-4.164	0.177	0.038	0.019	-4.183	-3.502	-0.681	-9.089	-1.246
2.543	-2.891	-3.388	-3.473	0.154	0.030	0.014	-3.487	-3.045	-0.442	-6.010	-0.690
2.743	-2.465	-2.801	-2.857	0.125	0.023	0.010	-2.867	-2.590	-0.277	-4.137	-0.369
2.944	-2.076	-2.284	-2.319	0.105	0.018	0.007	-2.326	-2.181	-0.146	-2.997	-0.196
3.144	-1.757	-1.869	-1.890	0.083	0.014	0.005	-1.895	-1.840	-0.055	-2.269	-0.101
3.346	-1.489	-1.541	-1.552	0.072	0.012	0.004	-1.556	-1.561	0.005	-1.791	-0.052
3.546	-1.280	-1.292	-1.297	0.057	0.009	0.003	-1.299	-1.336	0.037	-1.459	-0.026
3.750	-1.103	-1.089	-1.088	0.050	0.008	0.002	-1.091	-1.153	0.062	-1.220	-0.013
3.951	-0.959	-0.927	-0.924	0.038	0.006	0.002	-0.926	-0.997	0.071	-1.035	-0.007
4.160	-0.840	-0.799	-0.794	0.037	0.005	0.002	-0.796	-0.878	0.082	-0.901	-0.003
4.361	-0.741	-0.711	-0.707	0.029	0.004	0.001	-0.708	-0.770	0.062	-0.785	-0.002
4.561	-0.653	-0.622	-0.618	0.027	0.004	0.001	-0.619	-0.681	0.062	-0.691	-0.001
4.779	-0.586	-0.564	-0.560	0.024	0.003	0.001	-0.561	-0.610	0.049	-0.617	0.000
Conf2	-3.222	-4.285	-4.475	0.089	0.042	0.033	-4.508	-3.311	-1.196	-18.535	-3.029
Conf3	-2.608	-3.991	-4.227	0.143	0.036	0.019	-4.246	-2.751	-1.496	-16.433	-2.792
Conf4	-2.231	-3.047	-3.177	0.316	0.141	0.123	-3.300	-2.548	-0.752	-9.753	-1.499

$r_{O\cdots H}$	$\Delta E_{el-prep}^{HF}$	$\Delta E_{el-prep}^{HF,X}$	$\Delta E_{el-prep}^{HF,Y}$	$E_{elstat} + \Delta E_{el-prep}^{HF}$	ΔE_{int}^{C-WP}	$\Delta E_{int}^{C-(T)}$	ΔE_{int}^{C-SP}	$\Delta E_{int}^{C-SP,a}$	E_{res}^{C-SP}	E_{DISP}^{C-SP}	$E_{DISP}^{C-SP,a}$
PES	on Conf1										
1.743	41.301	20.699	20.602	3.907	-0.054	-0.311	-1.6697	-1.6695	0.024	-1.694	-1.690
1.943	22.811	11.588	11.223	0.068	-0.045	-0.220	-1.1602	-1.1601	0.028	-1.188	-1.185
2.144	12.592	6.498	6.095	-1.625	-0.035	-0.155	-0.8109	-0.8106	0.082	-0.893	-0.890
2.343	6.832	3.563	3.270	-2.256	-0.027	-0.105	-0.5494	-0.5492	0.084	-0.634	-0.631
2.543	3.655	1.918	1.737	-2.355	-0.020	-0.068	-0.3537	-0.3534	0.132	-0.486	-0.484
2.743	1.916	1.006	0.910	-2.221	-0.017	-0.042	-0.2179	-0.2177	0.136	-0.354	-0.352
2.944	1.013	0.533	0.480	-1.985	-0.011	-0.024	-0.1103	-0.1102	0.146	-0.256	-0.255
3.144	0.530	0.279	0.251	-1.739	-0.007	-0.012	-0.0367	-0.0366	0.152	-0.189	-0.188
3.346	0.283	0.149	0.134	-1.508	-0.004	-0.003	0.0120	0.0122	0.151	-0.139	-0.138
3.546	0.148	0.077	0.071	-1.310	-0.003	0.002	0.0377	0.0379	0.144	-0.106	-0.105
3.750	0.080	0.041	0.039	-1.140	-0.002	0.005	0.0583	0.0585	0.137	-0.079	-0.078
3.951	0.045	0.023	0.022	-0.990	-0.002	0.007	0.0662	0.0663	0.125	-0.059	-0.058
4.160	0.026	0.013	0.013	-0.875	-0.001	0.008	0.0746	0.0747	0.118	-0.044	-0.043
4.361	0.017	0.009	0.008	-0.769	-0.001	0.007	0.0560	0.0561	0.090	-0.034	-0.033
4.561	0.011	0.006	0.005	-0.680	-0.002	0.007	0.0558	0.0559	0.082	-0.026	-0.025
4.779	0.008	0.004	0.004	-0.609	-0.002	0.007	0.0441	0.0442	0.063	-0.019	-0.019
Conf2	18.253	8.900	9.353	-0.282	-0.048	-0.181	-0.9674		0.171	-1.139	
Conf3	16.474	8.237	8.237	0.041	-0.053	-0.219	-1.2236		0.085	-1.308	
Conf4	8.705	4.165	4.540	-1.048	-0.041	-0.112	-0.5992		0.347	-0.946	

[a] Without singles

2.2. aug-cc-pVQZ Energies

$r_{O\cdots H}$	ΔE_{HF}	ΔE_{CCSD}	$\Delta E_{CCSD(T)}$	$\Delta E_{geo-prep}$ HF	$\Delta E_{geo-prep}$ CCSD	$\Delta E_{geo-prep}$ CCSD(T)	ΔE_{int}	ΔE^{HF}_{int}	ΔE^{C}_{int}	E_{elstat}	E_{exch}
PES	on Conf1										
1.743	-2.006	-3.956	-4.306	0.449	0.206	0.167	-4.473	-2.455	-2.018	-37.440	-6.384
1.943	-3.482	-4.771	-5.008	0.207	0.079	0.058	-5.066	-3.689	-1.377	-22.806	-3.761
2.144	-3.603	-4.522	-4.692	0.207	0.068	0.047	-4.739	-3.810	-0.929	-14.257	-2.194
2.343	-3.292	-3.934	-4.052	0.187	0.057	0.038	-4.090	-3.478	-0.611	-9.102	-1.245
2.543	-2.853	-3.296	-3.375	0.162	0.047	0.030	-3.406	-3.015	-0.390	-6.011	-0.690
2.743	-2.432	-2.712	-2.762	0.131	0.037	0.023	-2.785	-2.563	-0.223	-4.132	-0.370
2.944	-2.050	-2.225	-2.256	0.110	0.030	0.018	-2.274	-2.160	-0.114	-2.989	-0.197
3.144	-1.739	-1.837	-1.855	0.087	0.023	0.014	-1.869	-1.826	-0.043	-2.260	-0.102
3.346	-1.475	-1.523	-1.533	0.075	0.019	0.011	-1.544	-1.550	0.005	-1.785	-0.053
3.546	-1.267	-1.291	-1.296	0.059	0.015	0.009	-1.304	-1.326	0.022	-1.454	-0.026
3.750	-1.091	-1.095	-1.095	0.052	0.013	0.008	-1.103	-1.143	0.041	-1.217	-0.013
3.951	-0.950	-0.937	-0.935	0.039	0.009	0.005	-0.941	-0.989	0.049	-1.034	-0.007
4.160	-0.834	-0.806	-0.802	0.039	0.009	0.005	-0.807	-0.872	0.065	-0.900	-0.003
4.361	-0.736	-0.701	-0.696	0.030	0.007	0.004	-0.700	-0.766	0.066	-0.784	-0.002
4.561	-0.648	-0.611	-0.606	0.028	0.007	0.004	-0.610	-0.677	0.067	-0.690	-0.001
4.779	-0.582	-0.544	-0.538	0.024	0.006	0.003	-0.542	-0.606	0.065	-0.616	0.000
Conf2	-3.220	-4.241	-4.427	0.097	0.053	0.043	-4.470	-3.317	-1.153	-18.588	-3.028
Conf3	-2.570	-3.937	-4.172	0.154	0.054	0.037	-4.209	-2.724	-1.486	-16.427	-2.791
Conf4	-2.210	-3.043	-3.175	0.319	0.156	0.137	-3.311	-2.529	-0.782	-9.751	-1.501

$r_{O\cdots H}$	$\Delta E^{HF}_{el-prep}$	$\Delta E^{HF,X}_{el-prep}$	$\Delta E^{HF,Y}_{el-prep}$	$E_{elstat} + \Delta E^{HF}_{el-prep}$	ΔE^{C-WP}_{int}	$\Delta E^{C-(T)}_{int}$	ΔE^{C-SP}_{int}	$\Delta E^{C-SP,a}_{int}$	E^{C-SP}_{res}	E^{C-SP}_{DISP}	$E^{C-SP,a}_{DISP}$
PES	on Conf1										
1.743	41.369	20.788	20.581	3.929	-0.082	-0.311	-1.6259	-1.6258	0.070	-1.696	-1.694
1.943	22.878	11.654	11.224	0.071	-0.063	-0.216	-1.0976	-1.0975	0.119	-1.217	-1.215
2.144	12.642	6.534	6.108	-1.616	-0.047	-0.149	-0.7326	-0.7324	0.143	-0.876	-0.874
2.343	6.869	3.586	3.283	-2.233	-0.036	-0.098	-0.4775	-0.4774	0.161	-0.638	-0.637
2.543	3.685	1.938	1.747	-2.326	-0.026	-0.062	-0.3027	-0.3025	0.168	-0.471	-0.469
2.743	1.939	1.023	0.916	-2.193	-0.019	-0.037	-0.1672	-0.1671	0.187	-0.354	-0.353
2.944	1.026	0.542	0.484	-1.963	-0.014	-0.020	-0.0808	-0.0807	0.191	-0.272	-0.271
3.144	0.536	0.282	0.254	-1.724	-0.008	-0.009	-0.0258	-0.0257	0.167	-0.192	-0.191
3.346	0.287	0.150	0.137	-1.497	-0.005	-0.002	0.0121	0.0123	0.153	-0.141	-0.140
3.546	0.154	0.080	0.075	-1.300	-0.003	0.002	0.0231	0.0232	0.129	-0.106	-0.105
3.750	0.087	0.044	0.043	-1.130	-0.002	0.005	0.0379	0.0380	0.117	-0.079	-0.078
3.951	0.051	0.026	0.025	-0.983	-0.001	0.006	0.0439	0.0440	0.103	-0.059	-0.059
4.160	0.031	0.015	0.016	-0.869	-0.001	0.008	0.0584	0.0585	0.102	-0.044	-0.043
4.361	0.020	0.009	0.011	-0.765	-0.001	0.008	0.0593	0.0594	0.093	-0.034	-0.033
4.561	0.014	0.006	0.007	-0.676	-0.002	0.008	0.0604	0.0605	0.086	-0.026	-0.025
4.779	0.010	0.004	0.006	-0.606	-0.002	0.008	0.0585	0.0586	0.078	-0.020	-0.019
Conf2	18.298	8.939	9.359	-0.289	-0.066	-0.177	-0.9105		0.205	-1.116	
Conf3	16.495	8.247	8.247	0.067	-0.070	-0.219	-1.1971		0.140	-1.337	
Conf4	8.722	4.183	4.539	-1.028	-0.051	-0.112	-0.6192		0.293	-0.912	

[a] Without singles

2.3. CBS-Only Corrected Energies

$r_{O\text{---}H}$	ΔE HF	ΔE CCSD	ΔE CCSD(T)	$\Delta E_{geo\text{-}prep}$ HF	$\Delta E_{geo\text{-}prep}$ CCSD	$\Delta E_{geo\text{-}prep}$ CCSD(T)	ΔE_{int}	ΔE_{int}^{HF}	ΔE_{int}^{C}	E_{elstat}	E_{exch}
PES	on Conf1										
1.743	-1.991	-3.917	-4.268	0.456	0.226	0.186	-4.453	-2.447	-2.006	-37.454	-6.382
1.943	-3.476	-4.727	-4.961	0.211	0.089	0.068	-5.029	-3.687	-1.342	-22.826	-3.760
2.144	-3.596	-4.461	-4.627	0.210	0.079	0.057	-4.684	-3.807	-0.877	-14.269	-2.194
2.343	-3.281	-3.872	-3.985	0.190	0.067	0.047	-4.033	-3.471	-0.562	-9.107	-1.245
2.543	-2.842	-3.246	-3.321	0.165	0.056	0.039	-3.360	-3.007	-0.353	-6.011	-0.690
2.743	-2.421	-2.662	-2.709	0.133	0.044	0.030	-2.738	-2.554	-0.184	-4.130	-0.370
2.944	-2.042	-2.193	-2.221	0.112	0.036	0.024	-2.245	-2.154	-0.091	-2.987	-0.197
3.144	-1.733	-1.821	-1.838	0.088	0.028	0.018	-1.856	-1.822	-0.034	-2.258	-0.102
3.346	-1.471	-1.516	-1.525	0.076	0.023	0.015	-1.541	-1.547	0.006	-1.783	-0.053
3.546	-1.263	-1.296	-1.300	0.060	0.018	0.012	-1.312	-1.323	0.011	-1.453	-0.026
3.750	-1.088	-1.104	-1.105	0.053	0.016	0.010	-1.115	-1.141	0.026	-1.216	-0.013
3.951	-0.947	-0.949	-0.947	0.040	0.012	0.007	-0.955	-0.987	0.032	-1.033	-0.007
4.160	-0.832	-0.814	-0.810	0.039	0.011	0.007	-0.817	-0.871	0.053	-0.899	-0.003
4.361	-0.734	-0.696	-0.690	0.031	0.009	0.006	-0.695	-0.765	0.069	-0.784	-0.002
4.561	-0.647	-0.605	-0.600	0.029	0.008	0.005	-0.605	-0.676	0.071	-0.689	-0.001
4.779	-0.581	-0.531	-0.525	0.025	0.007	0.004	-0.529	-0.605	0.076	-0.616	0.000
Conf2	-3.219	-4.210	-4.394	0.100	0.057	0.048	-4.441	-3.319	-1.122	-18.603	-3.028
Conf3	-2.558	-3.913	-4.149	0.157	0.062	0.045	-4.194	-2.715	-1.479	-16.426	-2.791
Conf4	-2.204	-3.049	-3.181	0.319	0.166	0.146	-3.327	-2.523	-0.804	-9.750	-1.501

$r_{O\text{---}H}$	$\Delta E_{el-prep}^{HF}$	$\Delta E_{el-prep}^{HF,X}$	$\Delta E_{el-prep}^{HF,Y}$	$E_{elstat} + \Delta E_{el-prep}^{HF}$	ΔE_{int}^{C-WP}	$\Delta E_{int}^{C-(T)}$	ΔE_{int}^{C-SP}	$\Delta E_{int}^{C-SP,a}$	E_{res}^{C-SP}	E_{DISP}^{C-SP}	$E_{DISP}^{C-SP,a}$
PES	on Conf1										
1.743	41.389	20.814	20.575	3.935	-0.101	-0.311	-1.5947	-1.5946	0.103	-1.698	-1.696
1.943	22.898	11.674	11.224	0.072	-0.076	-0.213	-1.0531	-1.0529	0.184	-1.237	-1.236
2.144	12.657	6.545	6.112	-1.613	-0.057	-0.144	-0.6768	-0.6768	0.187	-0.864	-0.863
2.343	6.880	3.593	3.288	-2.227	-0.042	-0.093	-0.4263	-0.4263	0.215	-0.642	-0.640
2.543	3.694	1.944	1.750	-2.317	-0.029	-0.058	-0.2664	-0.2663	0.193	-0.459	-0.459
2.743	1.946	1.028	0.918	-2.184	-0.020	-0.033	-0.1311	-0.1310	0.224	-0.355	-0.354
2.944	1.030	0.545	0.485	-1.957	-0.015	-0.016	-0.0597	-0.0596	0.223	-0.283	-0.282
3.144	0.538	0.283	0.255	-1.720	-0.009	-0.007	-0.0181	-0.0180	0.177	-0.195	-0.194
3.346	0.289	0.151	0.138	-1.494	-0.005	-0.001	0.0122	0.0123	0.154	-0.142	-0.142
3.546	0.156	0.080	0.076	-1.297	-0.003	0.002	0.0126	0.0127	0.119	-0.106	-0.105
3.750	0.089	0.045	0.044	-1.127	-0.002	0.005	0.0234	0.0235	0.102	-0.079	-0.079
3.951	0.053	0.026	0.026	-0.980	-0.001	0.006	0.0280	0.0281	0.088	-0.060	-0.059
4.160	0.032	0.015	0.017	-0.868	-0.002	0.008	0.0469	0.0470	0.091	-0.044	-0.043
4.361	0.021	0.009	0.011	-0.763	-0.001	0.009	0.0617	0.0618	0.096	-0.034	-0.034
4.561	0.014	0.006	0.008	-0.675	-0.002	0.009	0.0636	0.0638	0.089	-0.026	-0.026
4.779	0.011	0.005	0.006	-0.605	-0.002	0.009	0.0687	0.0688	0.089	-0.020	-0.020
Conf2	18.312	8.951	9.361	-0.291	-0.078	-0.174	-0.8700		0.230	-1.100	
Conf3	16.501	8.250	8.250	0.075	-0.082	-0.218	-1.1783		0.179	-1.358	
Conf4	8.728	4.189	4.539	-1.022	-0.058	-0.113	-0.6334		0.255	-0.889	

[a] Without singles

2.4. BSSE-Corrected aug-cc-pVTZ Energies

$r_{O\cdots H}$	ΔE *HF*	ΔE CCSD	ΔE CCSD(T)	$\Delta E_{geo-prep}$ *HF*	$\Delta E_{geo-prep}$ CCSD	$\Delta E_{geo-prep}$ CCSD(T)	ΔE_{int}	ΔE_{int}^{HF}	ΔE_{int}^{C}	E_{elstat}	E_{exch}
PES	on	Conf1									
1.743	-1.955	-3.521	-3.841	0.426	0.165	0.128	-3.969	-2.380	-1.588	-37.395	-6.389
1.943	-3.426	-4.454	-4.670	0.194	0.056	0.036	-4.707	-3.620	-1.086	-22.743	-3.763
2.144	-3.563	-4.311	-4.468	0.195	0.046	0.026	-4.493	-3.758	-0.735	-14.217	-2.196
2.343	-3.266	-3.791	-3.900	0.177	0.038	0.019	-3.918	-3.443	-0.475	-9.089	-1.246
2.543	-2.839	-3.198	-3.271	0.154	0.030	0.014	-3.285	-2.993	-0.292	-6.010	-0.690
2.743	-2.425	-2.658	-2.704	0.125	0.023	0.010	-2.713	-2.550	-0.163	-4.137	-0.369
2.944	-2.048	-2.193	-2.221	0.105	0.018	0.007	-2.228	-2.154	-0.075	-2.997	-0.196
3.144	-1.740	-1.820	-1.836	0.083	0.014	0.005	-1.841	-1.823	-0.018	-2.269	-0.101
3.346	-1.478	-1.512	-1.520	0.072	0.012	0.004	-1.524	-1.549	0.025	-1.791	-0.052
3.546	-1.270	-1.282	-1.284	0.057	0.009	0.003	-1.287	-1.327	0.039	-1.459	-0.026
3.750	-1.093	-1.089	-1.089	0.050	0.008	0.002	-1.091	-1.144	0.053	-1.220	-0.013
3.951	-0.951	-0.930	-0.927	0.038	0.006	0.002	-0.929	-0.989	0.060	-1.035	-0.007
4.160	-0.835	-0.799	-0.794	0.037	0.005	0.002	-0.796	-0.872	0.076	-0.901	-0.003
4.361	-0.737	-0.696	-0.691	0.029	0.004	0.001	-0.692	-0.766	0.074	-0.785	-0.002
4.561	-0.649	-0.610	-0.604	0.027	0.004	0.001	-0.605	-0.677	0.071	-0.691	-0.001
4.779	-0.583	-0.554	-0.549	0.024	0.003	0.001	-0.550	-0.607	0.057	-0.617	0.000
Conf2	-3.167	-3.973	-4.143	0.089	0.042	0.033	-4.176	-3.256	-0.919	-18.535	-3.029
Conf3	-2.532	-3.677	-3.893	0.143	0.036	0.019	-3.912	-2.675	-1.237	-16.433	-2.792
Conf4	-2.190	-2.896	-3.014	0.316	0.141	0.123	-3.136	-2.506	-0.631	-9.753	-1.499

$r_{O\cdots H}$	$\Delta E_{el-prep}^{HF}$	$\Delta E_{el-prep}^{HF,X}$	$\Delta E_{el-prep}^{HF,Y}$	$E_{elstat} + \Delta E_{el-prep}^{HF}$	ΔE_{int}^{C-WP}	$\Delta E_{int}^{C-(T)}$	ΔE_{int}^{C-SP}	E_{res}^{C-SP}	E_{DISP}^{C-SP}
PES	on Conf1								
1.743	41.404	20.761	20.643	4.009	-0.054	-0.282	-1.252	0.442	-1.694
1.943	22.885	11.634	11.251	0.143	-0.045	-0.196	-0.845	0.343	-1.188
2.144	12.655	6.540	6.115	-1.562	-0.035	-0.136	-0.565	0.328	-0.893
2.343	6.891	3.605	3.287	-2.197	-0.027	-0.090	-0.359	0.275	-0.634
2.543	3.707	1.956	1.751	-2.303	-0.020	-0.056	-0.215	0.271	-0.486
2.743	1.956	1.036	0.921	-2.181	-0.017	-0.033	-0.114	0.240	-0.354
2.944	1.040	0.552	0.488	-1.957	-0.011	-0.017	-0.047	0.210	-0.256
3.144	0.547	0.290	0.257	-1.722	-0.007	-0.007	-0.004	0.185	-0.189
3.346	0.294	0.155	0.139	-1.497	-0.004	0.000	0.030	0.169	-0.139
3.546	0.158	0.082	0.076	-1.301	-0.003	0.003	0.039	0.145	-0.106
3.750	0.089	0.046	0.044	-1.131	-0.002	0.006	0.049	0.127	-0.079
3.951	0.053	0.027	0.026	-0.982	-0.002	0.007	0.055	0.114	-0.059
4.160	0.032	0.015	0.017	-0.869	-0.001	0.009	0.069	0.112	-0.044
4.361	0.021	0.010	0.011	-0.764	-0.001	0.008	0.066	0.100	-0.034
4.561	0.015	0.007	0.008	-0.676	-0.002	0.008	0.065	0.090	-0.026
4.779	0.011	0.005	0.006	-0.606	-0.002	0.008	0.051	0.070	-0.019
Conf2	18.307	8.927	9.380	-0.228	-0.048	-0.161	-0.710	0.428	-1.139
Conf3	16.550	8.275	8.275	0.117	-0.053	-0.199	-0.985	0.323	-1.308
Conf4	8.746	4.193	4.553	-1.006	-0.041	-0.100	-0.490	0.456	-0.946

2.5. BSSE-Corrected aug-cc-pVQZ Energies

$r_{O\text{---}H}$	ΔE HF	ΔE CCSD	ΔE CCSD(T)	$\Delta E_{geo\text{-}prep}$ HF	$\Delta E_{geo\text{-}prep}$ CCSD	$\Delta E_{geo\text{-}prep}$ CCSD(T)	ΔE_{int}	ΔE_{int}^{HF}	ΔE_{int}^{C}	E_{elstat}	E_{exch}
PES	on	Conf1									
1.743	-1.969	-4.609	-4.066	0.449	0.206	0.167	-4.233	-2.418	-1.815	-37.440	-6.384
1.943	-3.452	-4.403	-4.838	0.207	0.079	0.058	-4.896	-3.659	-1.236	-22.806	-3.761
2.144	-3.578	-3.837	-4.567	0.207	0.068	0.047	-4.614	-3.784	-0.830	-14.257	-2.194
2.343	-3.269	-3.232	-3.949	0.187	0.057	0.038	-3.987	-3.456	-0.531	-9.102	-1.245
2.543	-2.835	-2.672	-3.308	0.162	0.047	0.030	-3.338	-2.997	-0.341	-6.011	-0.690
2.743	-2.418	-2.201	-2.720	0.131	0.037	0.023	-2.743	-2.549	-0.194	-4.132	-0.370
2.944	-2.039	-1.824	-2.230	0.110	0.030	0.018	-2.248	-2.149	-0.099	-2.989	-0.197
3.144	-1.730	-1.511	-1.841	0.087	0.023	0.014	-1.855	-1.817	-0.038	-2.260	-0.102
3.346	-1.469	-1.285	-1.520	0.075	0.019	0.011	-1.532	-1.544	0.012	-1.785	-0.053
3.546	-1.263	-1.093	-1.289	0.059	0.015	0.009	-1.298	-1.322	0.024	-1.454	-0.026
3.750	-1.088	-0.940	-1.093	0.052	0.013	0.008	-1.101	-1.140	0.040	-1.217	-0.013
3.951	-0.947	-0.802	-0.938	0.039	0.009	0.005	-0.943	-0.987	0.044	-1.034	-0.007
4.160	-0.832	-0.705	-0.798	0.039	0.009	0.005	-0.803	-0.870	0.067	-0.900	-0.003
4.361	-0.734	-0.617	-0.700	0.030	0.007	0.004	-0.704	-0.764	0.061	-0.784	-0.002
4.561	-0.647	-0.539	-0.612	0.028	0.007	0.004	-0.615	-0.675	0.060	-0.690	-0.001
4.779	-0.581	-4.609	-0.533	0.024	0.006	0.003	-0.536	-0.606	0.069	-0.616	0.000
Conf2	-3.194	-4.108	-4.286	0.097	0.053	0.043	-4.330	-3.291	-1.039	-18.588	-3.028
Conf3	-2.539	-3.816	-4.046	0.154	0.054	0.037	-4.083	-2.693	-1.390	-16.427	-2.791
Conf4	-2.189	-2.973	-3.100	0.319	0.156	0.137	-3.237	-2.507	-0.729	-9.751	-1.501

$r_{O\text{---}H}$	$\Delta E_{el-prep}^{HF}$	$\Delta E_{el-prep}^{HF,X}$	$\Delta E_{el-prep}^{HF,Y}$	$E_{elstat} + \Delta E_{el-prep}^{HF}$	ΔE_{int}^{C-WP}	$\Delta E_{int}^{C-(T)}$	ΔE_{int}^{C-SP}	E_{res}^{C-SP}	E_{DISP}^{C-SP}
PES	on Conf1								
1.743	41.406	20.812	20.593	3.966	-0.082	-0.298	-1.435	3.966	-1.696
1.943	22.908	11.674	11.234	0.101	-0.063	-0.208	-0.966	0.101	-1.217
2.144	12.667	6.552	6.115	-1.590	-0.047	-0.143	-0.639	-1.590	-0.876
2.343	6.891	3.602	3.289	-2.211	-0.036	-0.093	-0.402	-2.211	-0.638
2.543	3.703	1.952	1.752	-2.308	-0.026	-0.059	-0.256	-2.308	-0.471
2.743	1.953	1.033	0.920	-2.179	-0.019	-0.034	-0.141	-2.179	-0.354
2.944	1.037	0.550	0.487	-1.952	-0.014	-0.018	-0.068	-1.952	-0.272
3.144	0.545	0.288	0.256	-1.716	-0.008	-0.008	-0.022	-1.716	-0.192
3.346	0.294	0.155	0.139	-1.491	-0.005	-0.001	0.018	-1.491	-0.141
3.546	0.158	0.082	0.076	-1.296	-0.003	0.003	0.025	-1.296	-0.106
3.750	0.090	0.046	0.044	-1.127	-0.002	0.005	0.037	-1.127	-0.079
3.951	0.053	0.027	0.026	-0.980	-0.001	0.006	0.039	-0.980	-0.059
4.160	0.032	0.016	0.017	-0.867	-0.001	0.008	0.060	-0.867	-0.044
4.361	0.022	0.010	0.011	-0.763	-0.001	0.008	0.054	-0.763	-0.034
4.561	0.015	0.007	0.008	-0.675	-0.002	0.008	0.054	-0.675	-0.026
4.779	0.011	0.005	0.006	-0.605	-0.002	0.009	0.062	-0.605	-0.020
Conf2	18.325	8.956	9.369	-0.263	-0.066	-0.169	-0.804	-0.263	-1.116
Conf3	16.526	8.263	8.263	0.098	-0.070	-0.213	-1.107	0.098	-1.337
Conf4	8.744	4.195	4.548	-1.007	-0.051	-0.108	-0.571	-1.007	-0.912

2.6. BSSE-Followed CBS Energies

$r_{O\text{---}H}$	ΔE HF	ΔE CCSD	ΔE CCSD(T)	$\Delta E_{geo\text{-}prep}$ HF	$\Delta E_{geo\text{-}prep}$ CCSD	$\Delta E_{geo\text{-}prep}$ CCSD(T)	ΔE_{int}	ΔE_{int}^{HF}	ΔE_{int}^{C}	E_{elstat}	E_{exch}
PES	on	Conf1									
1.743	-1.973	-3.870	-4.220	0.456	0.226	0.186	-4.406	-2.429	-1.976	-37.454	-6.382
1.943	-3.460	-4.709	-4.946	0.211	0.089	0.068	-5.014	-3.671	-1.343	-22.826	-3.760
2.144	-3.582	-4.462	-4.631	0.210	0.079	0.057	-4.689	-3.792	-0.897	-14.269	-2.194
2.343	-3.270	-3.868	-3.984	0.190	0.067	0.047	-4.031	-3.460	-0.571	-9.107	-1.245
2.543	-2.834	-3.257	-3.336	0.165	0.056	0.039	-3.375	-2.999	-0.376	-6.011	-0.690
2.743	-2.416	-2.685	-2.735	0.133	0.044	0.030	-2.765	-2.549	-0.216	-4.130	-0.370
2.944	-2.036	-2.210	-2.241	0.112	0.036	0.024	-2.265	-2.148	-0.117	-2.987	-0.197
3.144	-1.727	-1.831	-1.849	0.088	0.028	0.018	-1.867	-1.816	-0.052	-2.258	-0.102
3.346	-1.466	-1.515	-1.524	0.076	0.023	0.015	-1.539	-1.542	0.002	-1.783	-0.053
3.546	-1.261	-1.291	-1.295	0.060	0.018	0.012	-1.307	-1.321	0.014	-1.453	-0.026
3.750	-1.086	-1.098	-1.099	0.053	0.016	0.010	-1.109	-1.140	0.031	-1.216	-0.013
3.951	-0.946	-0.948	-0.947	0.040	0.012	0.007	-0.954	-0.986	0.032	-1.033	-0.007
4.160	-0.831	-0.806	-0.802	0.039	0.011	0.007	-0.809	-0.870	0.061	-0.899	-0.003
4.361	-0.734	-0.712	-0.707	0.031	0.009	0.006	-0.713	-0.764	0.051	-0.784	-0.002
4.561	-0.646	-0.623	-0.618	0.029	0.008	0.005	-0.623	-0.675	0.052	-0.689	-0.001
4.779	-0.580	-0.529	-0.523	0.025	0.007	0.004	-0.527	-0.605	0.078	-0.616	0.000
Conf2	-3.202	-4.193	-4.377	0.100	0.057	0.048	-4.425	-3.302	-1.124	-18.603	-3.028
Conf3	-2.541	-3.911	-4.152	0.157	0.062	0.045	-4.197	-2.698	-1.499	-16.426	-2.791
Conf4	-2.189	-3.028	-3.161	0.319	0.166	0.146	-3.307	-2.508	-0.800	-9.750	-1.501

$r_{O\text{---}H}$	$\Delta E_{el-prep}^{HF}$	$\Delta E_{el-prep}^{HF,X}$	$\Delta E_{el-prep}^{HF,Y}$	$E_{elstat} + \Delta E_{el-prep}^{HF}$	ΔE_{int}^{C-WP}	$\Delta E_{int}^{C-(T)}$	ΔE_{int}^{C-SP}	E_{res}^{C-SP}	E_{DISP}^{C-SP}
PES	on Conf1								
1.743	41.406	20.828	20.578	3.953	-0.101	-0.310	-1.565	0.132	-1.698
1.943	22.915	11.686	11.229	0.089	-0.076	-0.216	-1.052	0.185	-1.237
2.144	12.671	6.556	6.115	-1.598	-0.057	-0.147	-0.693	0.171	-0.864
2.343	6.891	3.601	3.290	-2.215	-0.042	-0.096	-0.433	0.209	-0.642
2.543	3.702	1.950	1.752	-2.309	-0.029	-0.062	-0.285	0.174	-0.459
2.743	1.952	1.032	0.920	-2.179	-0.020	-0.036	-0.160	0.194	-0.355
2.944	1.036	0.549	0.487	-1.951	-0.015	-0.019	-0.082	0.200	-0.283
3.144	0.544	0.288	0.256	-1.714	-0.009	-0.008	-0.034	0.161	-0.195
3.346	0.293	0.155	0.139	-1.489	-0.005	-0.001	0.009	0.151	-0.142
3.546	0.158	0.082	0.076	-1.294	-0.003	0.002	0.015	0.121	-0.106
3.750	0.090	0.046	0.044	-1.126	-0.002	0.005	0.028	0.107	-0.079
3.951	0.054	0.027	0.026	-0.979	-0.001	0.006	0.027	0.087	-0.060
4.160	0.033	0.016	0.017	-0.867	-0.002	0.008	0.055	0.098	-0.044
4.361	0.022	0.010	0.011	-0.763	-0.001	0.008	0.044	0.078	-0.034
4.561	0.015	0.007	0.008	-0.674	-0.002	0.008	0.046	0.072	-0.026
4.779	0.011	0.005	0.006	-0.605	-0.002	0.009	0.071	0.090	-0.020
Conf2	18.330	8.964	9.366	-0.274	-0.078	-0.175	-0.870	0.229	-1.100
Conf3	16.518	8.259	8.259	0.093	-0.082	-0.223	-1.194	0.164	-1.358
Conf4	8.743	4.196	4.547	-1.007	-0.058	-0.113	-0.628	0.260	-0.889

3. SAPT Energies of W---W (kcal/mol)

3.1. HF-SAPT Energies

	aug-cc-pVTZ						aug-cc-pVQZ					
$r_{O\text{---}H}$	ΔE_{int}	E_{pol}	E_{exch}	E_{ind}	$E_{exch\text{-}ind}$	$\delta(HF)$	ΔE_{int}	E_{pol}	E_{exch}	E_{ind}	$E_{exch\text{-}ind}$	$\delta(HF)$
1.743	-2.382	-12.357	14.411	-6.211	3.733	-1.958	-2.418	-12.363	14.395	-6.217	3.731	-1.966
1.943	-3.622	-8.369	7.048	-3.017	1.648	-0.932	-3.660	-8.382	7.034	-3.010	1.635	-0.937
2.144	-3.760	-5.957	3.448	-1.537	0.734	-0.447	-3.785	-5.969	3.442	-1.535	0.727	-0.450
2.343	-3.445	-4.409	1.668	-0.810	0.320	-0.214	-3.456	-4.416	1.666	-0.812	0.320	-0.215
2.543	-2.994	-3.385	0.800	-0.446	0.138	-0.102	-2.998	-3.386	0.799	-0.449	0.141	-0.102
2.743	-2.551	-2.680	0.376	-0.257	0.058	-0.049	-2.549	-2.677	0.376	-0.259	0.060	-0.049
2.944	-2.155	-2.177	0.178	-0.157	0.025	-0.024	-2.150	-2.171	0.177	-0.158	0.026	-0.024
3.144	-1.824	-1.804	0.082	-0.101	0.011	-0.012	-1.818	-1.798	0.082	-0.101	0.011	-0.012
3.346	-1.550	-1.519	0.038	-0.068	0.005	-0.006	-1.544	-1.513	0.038	-0.068	0.005	-0.006
3.546	-1.327	-1.296	0.017	-0.048	0.002	-0.003	-1.322	-1.291	0.017	-0.048	0.002	-0.003
3.750	-1.144	-1.117	0.008	-0.034	0.001	-0.002	-1.141	-1.113	0.008	-0.034	0.001	-0.002
3.951	-0.989	-0.967	0.004	-0.025	0.000	-0.001	-0.987	-0.965	0.004	-0.025	0.000	-0.001
4.160	-0.872	-0.854	0.002	-0.019	0.000	-0.001	-0.870	-0.852	0.002	-0.019	0.000	-0.001
4.361	-0.766	-0.751	0.001	-0.015	0.000	0.000	-0.765	-0.750	0.001	-0.015	0.000	0.000
4.561	-0.677	-0.665	0.000	-0.012	0.000	0.000	-0.675	-0.664	0.000	-0.012	0.000	0.000
4.779	-0.607	-0.597	0.000	-0.009	0.000	0.000	-0.606	-0.596	0.000	-0.009	0.000	0.000

3.2. DFT-SAPT/aug-cc-pVTZ Energies

$r_{O\text{---}H}$	E_{pol}	E_{exch}	E_{ind}	$E_{exch\text{-}ind}$	$\delta(HF)$	$E_{no\text{-}disp}$	$E_{disp(genuine)}$	$E_{exch\text{-}disp}$	E_{disp} [a]	ΔE_{int}
1.743	-12.069	16.031	-6.760	4.443	-1.958	-0.313	-4.222	0.973	-3.249	-3.562
1.943	-8.077	8.017	-3.276	2.006	-0.932	-2.262	-2.694	0.544	-2.150	-4.412
2.144	-5.683	4.016	-1.658	0.913	-0.447	-2.859	-1.748	0.302	-1.446	-4.305
2.343	-4.153	1.988	-0.862	0.408	-0.214	-2.832	-1.143	0.164	-0.978	-3.811
2.543	-3.150	0.973	-0.464	0.181	-0.102	-2.562	-0.754	0.088	-0.666	-3.228
2.743	-2.467	0.466	-0.260	0.078	-0.049	-2.231	-0.501	0.046	-0.455	-2.686
2.944	-1.988	0.225	-0.154	0.034	-0.024	-1.907	-0.339	0.024	-0.315	-2.222
3.144	-1.637	0.106	-0.096	0.015	-0.012	-1.624	-0.232	0.012	-0.220	-1.844
3.346	-1.372	0.051	-0.063	0.007	-0.006	-1.384	-0.162	0.006	-0.156	-1.540
3.546	-1.165	0.023	-0.043	0.003	-0.003	-1.185	-0.115	0.003	-0.112	-1.297
3.750	-1.001	0.011	-0.031	0.001	-0.002	-1.021	-0.083	0.002	-0.082	-1.103
3.951	-0.865	0.005	-0.022	0.001	-0.001	-0.883	-0.062	0.001	-0.061	-0.944
4.160	-0.761	0.002	-0.017	0.000	-0.001	-0.776	-0.046	0.000	-0.045	-0.821
4.361	-0.669	0.001	-0.013	0.000	0.000	-0.681	-0.035	0.000	-0.035	-0.716
4.561	-0.592	0.001	-0.010	0.000	0.000	-0.601	-0.027	0.000	-0.027	-0.629
4.779	-0.530	0.000	-0.008	0.000	0.000	-0.538	-0.021	0.000	-0.021	-0.559

[a] Sum of the genuine dispersion and exchange-dispersion energies

3.3. DFT-SAPT/aug-cc-pVQZ and DFT-SAPT/CBS Energies

r_{O---H}	E_{pol}	E_{exch}	E_{ind}	$E_{exch\text{-}ind}$	$\delta(HF)$	$E_{no\text{-}disp}$	$E_{disp(genuine)}$	$E_{exch\text{-}disp}$	E_{disp} [a]	ΔE_{int}	$E^{b}_{disp(genuine)}$	$E^{b}_{exch-disp}$	E_{dis} [a,b]	ΔE_{int} [b]
1.743	-12.072	16.021	-6.769	4.444	-1.966	-0.340	-4.414	1.031	-3.382	-3.723	-4.550	1.073	-3.477	-3.818
1.943	-8.088	8.009	-3.271	1.995	-0.937	-2.291	-2.814	0.579	-2.235	-4.526	-2.899	0.603	-2.296	-4.587
2.144	-5.693	4.016	-1.658	0.909	-0.450	-2.876	-1.822	0.322	-1.500	-4.376	-1.875	0.337	-1.538	-4.414
2.343	-4.159	1.989	-0.865	0.409	-0.215	-2.840	-1.188	0.176	-1.012	-3.852	-1.220	0.184	-1.036	-3.876
2.543	-3.151	0.975	-0.468	0.183	-0.102	-2.564	-0.782	0.095	-0.687	-3.251	-0.801	0.099	-0.702	-3.266
2.743	-2.466	0.468	-0.262	0.080	-0.049	-2.229	-0.518	0.049	-0.468	-2.697	-0.529	0.052	-0.477	-2.707
2.944	-1.984	0.226	-0.156	0.036	-0.024	-1.903	-0.350	0.026	-0.324	-2.227	-0.357	0.027	-0.330	-2.233
3.144	-1.633	0.106	-0.097	0.015	-0.012	-1.620	-0.239	0.013	-0.226	-1.845	-0.244	0.014	-0.230	-1.849
3.346	-1.368	0.051	-0.064	0.007	-0.006	-1.380	-0.167	0.007	-0.161	-1.540	-0.171	0.007	-0.164	-1.543
3.546	-1.162	0.023	-0.044	0.003	-0.003	-1.183	-0.118	0.003	-0.115	-1.298	-0.121	0.004	-0.117	-1.300
3.750	-0.999	0.011	-0.031	0.001	-0.002	-1.020	-0.086	0.002	-0.085	-1.104	-0.088	0.002	-0.086	-1.106
3.951	-0.865	0.005	-0.023	0.001	-0.001	-0.882	-0.064	0.001	-0.063	-0.945	-0.065	0.001	-0.064	-0.947
4.160	-0.761	0.002	-0.017	0.000	-0.001	-0.776	-0.047	0.000	-0.047	-0.823	-0.049	0.000	-0.048	-0.824
4.361	-0.669	0.001	-0.013	0.000	0.000	-0.681	-0.036	0.000	-0.036	-0.717	-0.037	0.000	-0.037	-0.718
4.561	-0.592	0.001	-0.010	0.000	0.000	-0.602	-0.028	0.000	-0.028	-0.630	-0.029	0.000	-0.029	-0.630
4.779	-0.530	0.000	-0.008	0.000	0.000	-0.538	-0.022	0.000	-0.022	-0.560	-0.022	0.000	-0.022	-0.560

[a] Sum of the genuine dispersion and exchange-dispersion energies

[b] CBS-Corrected Energies

4. DLPNO-CCSD(T)/LED Energies of HF---HF (kcal/mol)

4.1. aug-cc-pVTZ Energies

r_{F---H}	ΔE HF	ΔE CCSD	ΔE CCSD(T)	$\Delta E_{geo-prep}$ HF	$\Delta E_{geo-prep}$ CCSD	$\Delta E_{geo-prep}$ CCSD(T)	ΔE_{int}	ΔE_{int}^{HF}	ΔE_{int}^{C}	E_{elstat}	E_{exch}
PES	on	Conf1									
1.627	-2.322	-3.817	-4.071	0.654	0.188	0.132	-4.203	-2.976	-1.227	-36.285	-5.754
1.827	-3.561	-4.570	-4.738	0.346	0.079	0.046	-4.784	-3.907	-0.876	-21.063	-3.188
2.026	-3.561	-4.285	-4.400	0.289	0.063	0.036	-4.436	-3.850	-0.585	-12.398	-1.682
2.227	-3.184	-3.686	-3.761	0.234	0.049	0.026	-3.787	-3.418	-0.369	-7.594	-0.856
2.427	-2.725	-3.060	-3.106	0.186	0.037	0.019	-3.125	-2.911	-0.214	-4.924	-0.423
2.627	-2.301	-2.499	-2.526	0.148	0.028	0.013	-2.540	-2.448	-0.091	-3.413	-0.203
2.828	-1.943	-2.041	-2.055	0.118	0.021	0.010	-2.065	-2.061	-0.004	-2.527	-0.095
3.028	-1.655	-1.686	-1.691	0.094	0.016	0.007	-1.698	-1.749	0.051	-1.979	-0.043
3.227	-1.426	-1.414	-1.412	0.075	0.013	0.005	-1.418	-1.501	0.083	-1.616	-0.019
3.430	-1.227	-1.200	-1.198	0.067	0.011	0.004	-1.202	-1.295	0.093	-1.355	-0.009
3.623	-1.097	-1.042	-1.036	0.050	0.008	0.003	-1.039	-1.147	0.107	-1.175	-0.003
3.823	-0.964	-0.911	-0.904	0.042	0.007	0.002	-0.907	-1.007	0.100	-1.023	-0.001
4.023	-0.852	-0.800	-0.794	0.036	0.006	0.002	-0.796	-0.888	0.092	-0.899	-0.001
4.223	-0.756	-0.707	-0.701	0.032	0.005	0.002	-0.703	-0.788	0.085	-0.795	0.000
4.424	-0.674	-0.628	-0.622	0.028	0.004	0.002	-0.623	-0.702	0.078	-0.707	0.000
4.624	-0.603	-0.560	-0.554	0.025	0.004	0.001	-0.556	-0.628	0.073	-0.632	0.000
4.824	-0.542	-0.501	-0.494	0.022	0.003	0.001	-0.495	-0.565	0.069	-0.568	0.000
Conf2	-3.172	-3.633	-3.714	0.154	0.029	0.014	-3.728	-3.326	-0.402	-12.444	-1.754
Conf3	-2.559	-3.512	-3.664	0.297	0.063	0.034	-3.699	-2.856	-0.842	-14.468	-2.212

r_{F---H}	$\Delta E_{el-prep}^{HF}$	$\Delta E_{el-prep}^{HF,X}$	$\Delta E_{el-prep}^{HF,Y}$	$E_{elstat} + \Delta E_{el-prep}^{HF}$	ΔE_{int}^{C-WP}	$\Delta E_{int}^{C-(T)}$	ΔE_{int}^{C-SP}	E_{res}^{C-SP}	E_{DISP}^{C-SP}
PES	on Conf1								
1.627	39.063	19.352	19.712	2.778	-0.058	-0.198	-0.972	0.379	-1.350
1.827	20.344	10.234	10.110	-0.719	-0.045	-0.136	-0.695	0.279	-0.975
2.026	10.230	5.216	5.014	-2.168	-0.032	-0.087	-0.466	0.191	-0.657
2.227	5.033	2.605	2.428	-2.561	-0.025	-0.052	-0.293	0.182	-0.475
2.427	2.436	1.274	1.163	-2.488	-0.020	-0.028	-0.166	0.161	-0.328
2.627	1.168	0.627	0.541	-2.245	-0.011	-0.012	-0.067	0.151	-0.218
2.828	0.562	0.316	0.246	-1.966	-0.006	-0.002	0.004	0.158	-0.154
3.028	0.273	0.163	0.111	-1.705	-0.004	0.004	0.050	0.153	-0.103
3.227	0.134	0.085	0.049	-1.482	-0.003	0.009	0.077	0.156	-0.079
3.430	0.069	0.047	0.022	-1.286	-0.002	0.009	0.085	0.144	-0.059
3.623	0.031	0.024	0.007	-1.144	-0.003	0.011	0.099	0.141	-0.042
3.823	0.018	0.016	0.002	-1.005	-0.002	0.011	0.092	0.123	-0.031
4.023	0.012	0.011	0.000	-0.887	-0.003	0.010	0.085	0.107	-0.023
4.223	0.008	0.009	-0.001	-0.787	-0.003	0.009	0.078	0.095	-0.016
4.424	0.006	0.006	-0.001	-0.702	-0.003	0.009	0.073	0.085	-0.012
4.624	0.004	0.005	-0.001	-0.628	-0.002	0.008	0.067	0.077	-0.010
4.824	0.003	0.004	0.000	-0.565	-0.002	0.009	0.062	0.069	-0.007
Conf2	10.872	5.268	5.604	-1.572	-0.045	-0.065	-0.291	0.500	-0.791
Conf3	13.824	6.912	6.912	-0.645	-0.052	-0.124	-0.666	0.266	-0.931

4.2. aug-cc-pVQZ Energies

$r_{F\text{---}H}$	ΔE HF	ΔE CCSD	ΔE CCSD(T)	$\Delta E_{geo\text{-}prep}$ HF	$\Delta E_{geo\text{-}prep}$ CCSD	$\Delta E_{geo\text{-}prep}$ CCSD(T)	ΔE_{int}	ΔE_{int}^{HF}	ΔE_{int}^{C}	E_{elstat}	E_{exch}
PES	on	Conf1									
1.627	-2.296	-3.749	-4.004	0.694	0.263	0.204	-4.207	-2.991	-1.216	-36.452	-5.753
1.827	-3.550	-4.487	-4.654	0.370	0.122	0.088	-4.742	-3.921	-0.821	-21.187	-3.189
2.026	-3.526	-4.161	-4.273	0.309	0.100	0.071	-4.344	-3.835	-0.509	-12.448	-1.682
2.227	-3.135	-3.544	-3.615	0.251	0.079	0.055	-3.670	-3.386	-0.284	-7.603	-0.856
2.427	-2.680	-2.929	-2.970	0.200	0.061	0.042	-3.012	-2.880	-0.132	-4.921	-0.423
2.627	-2.266	-2.402	-2.425	0.159	0.047	0.032	-2.457	-2.424	-0.033	-3.407	-0.203
2.828	-1.919	-1.987	-1.999	0.126	0.037	0.025	-2.023	-2.045	0.022	-2.519	-0.095
3.028	-1.637	-1.655	-1.658	0.101	0.029	0.019	-1.678	-1.738	0.060	-1.971	-0.043
3.227	-1.410	-1.391	-1.390	0.081	0.023	0.015	-1.405	-1.491	0.086	-1.610	-0.019
3.430	-1.212	-1.193	-1.191	0.072	0.020	0.013	-1.204	-1.284	0.080	-1.351	-0.009
3.623	-1.084	-1.029	-1.022	0.053	0.015	0.010	-1.031	-1.137	0.106	-1.172	-0.003
3.823	-0.953	-0.903	-0.896	0.045	0.012	0.008	-0.904	-0.999	0.095	-1.021	-0.001
4.023	-0.843	-0.804	-0.798	0.039	0.011	0.007	-0.804	-0.882	0.077	-0.897	-0.001
4.223	-0.748	-0.716	-0.710	0.034	0.009	0.006	-0.716	-0.782	0.066	-0.794	0.000
4.424	-0.667	-0.638	-0.633	0.030	0.008	0.005	-0.638	-0.697	0.060	-0.706	0.000
4.624	-0.597	-0.572	-0.567	0.027	0.007	0.005	-0.571	-0.624	0.053	-0.631	0.000
4.824	-0.537	-0.514	-0.509	0.024	0.007	0.004	-0.514	-0.561	0.048	-0.566	0.000
Conf2	-3.183	-3.600	-3.674	0.165	0.049	0.034	-3.708	-3.348	-0.360	-12.530	-1.755
Conf3	-2.473	-3.419	-3.577	0.318	0.101	0.071	-3.648	-2.791	-0.857	-14.471	-2.214

$r_{F\text{---}H}$	$\Delta E_{el-prep}^{HF}$	$\Delta E_{el-prep}^{HF,X}$	$\Delta E_{el-prep}^{HF,Y}$	$E_{elstat} + \Delta E_{el-prep}^{HF}$	ΔE_{int}^{C-WP}	$\Delta E_{int}^{C-(T)}$	ΔE_{int}^{C-SP}	E_{res}^{C-SP}	E_{DISP}^{C-SP}
PES	on Conf1								
1.627	39.214	19.526	19.688	2.762	-0.085	-0.195	-0.936	0.480	-1.416
1.827	20.455	10.347	10.109	-0.731	-0.064	-0.133	-0.624	0.332	-0.955
2.026	10.295	5.276	5.018	-2.153	-0.045	-0.083	-0.381	0.280	-0.661
2.227	5.073	2.641	2.432	-2.529	-0.031	-0.048	-0.206	0.258	-0.463
2.427	2.464	1.298	1.166	-2.457	-0.024	-0.023	-0.086	0.250	-0.335
2.627	1.185	0.640	0.545	-2.221	-0.014	-0.008	-0.011	0.221	-0.232
2.828	0.570	0.318	0.252	-1.950	-0.009	0.000	0.030	0.198	-0.168
3.028	0.277	0.161	0.116	-1.694	-0.005	0.006	0.059	0.178	-0.118
3.227	0.139	0.086	0.053	-1.471	-0.003	0.009	0.079	0.159	-0.080
3.430	0.075	0.050	0.026	-1.276	-0.002	0.010	0.073	0.129	-0.056
3.623	0.038	0.027	0.011	-1.134	-0.002	0.012	0.095	0.136	-0.041
3.823	0.024	0.018	0.006	-0.997	-0.002	0.012	0.086	0.116	-0.030
4.023	0.016	0.012	0.004	-0.881	-0.003	0.011	0.070	0.092	-0.022
4.223	0.011	0.009	0.003	-0.782	-0.003	0.010	0.060	0.077	-0.017
4.424	0.008	0.007	0.002	-0.697	-0.003	0.009	0.054	0.066	-0.012
4.624	0.007	0.005	0.001	-0.624	-0.003	0.008	0.047	0.057	-0.010
4.824	0.005	0.004	0.001	-0.561	-0.002	0.007	0.042	0.049	-0.007
Conf2	10.937	5.331	5.606	-1.593	-0.060	-0.058	-0.242	0.552	-0.794
Conf3	13.894	6.948	6.947	-0.577	-0.066	-0.128	-0.662	0.286	-0.948

4.3. CBS-Only Corrected Energies

r_{F---H}	ΔE HF	ΔE CCSD	ΔE CCSD(T)	$\Delta E_{geo-prep}$ HF	$\Delta E_{geo-prep}$ CCSD	$\Delta E_{geo-prep}$ CCSD(T)	ΔE_{int}	ΔE_{int}^{HF}	ΔE_{int}^{C}	E_{elstat}	E_{exch}
PES	On	Conf1									
1.627	-2.289	-3.711	-3.966	0.707	0.299	0.238	-4.204	-2.995	-1.209	-36.502	-5.752
1.827	-3.547	-4.432	-4.598	0.377	0.143	0.108	-4.706	-3.925	-0.782	-21.224	-3.189
2.026	-3.515	-4.088	-4.198	0.315	0.117	0.088	-4.286	-3.831	-0.455	-12.462	-1.682
2.227	-3.120	-3.462	-3.531	0.256	0.093	0.069	-3.600	-3.376	-0.224	-7.605	-0.856
2.427	-2.667	-2.853	-2.891	0.204	0.073	0.053	-2.945	-2.871	-0.074	-4.920	-0.423
2.627	-2.255	-2.347	-2.367	0.162	0.057	0.041	-2.408	-2.417	0.009	-3.405	-0.203
2.828	-1.911	-1.958	-1.968	0.129	0.045	0.032	-2.000	-2.040	0.040	-2.517	-0.095
3.028	-1.631	-1.640	-1.643	0.103	0.035	0.025	-1.668	-1.734	0.067	-1.969	-0.043
3.227	-1.405	-1.381	-1.380	0.083	0.028	0.020	-1.400	-1.487	0.087	-1.609	-0.019
3.430	-1.207	-1.195	-1.192	0.074	0.025	0.018	-1.210	-1.281	0.071	-1.350	-0.009
3.623	-1.080	-1.025	-1.017	0.055	0.018	0.013	-1.030	-1.134	0.104	-1.171	-0.003
3.823	-0.950	-0.902	-0.894	0.046	0.015	0.011	-0.905	-0.996	0.091	-1.021	-0.001
4.023	-0.840	-0.811	-0.804	0.040	0.013	0.009	-0.813	-0.880	0.067	-0.897	-0.001
4.223	-0.746	-0.725	-0.719	0.035	0.011	0.008	-0.727	-0.781	0.054	-0.793	0.000
4.424	-0.665	-0.649	-0.643	0.031	0.010	0.007	-0.650	-0.696	0.046	-0.705	0.000
4.624	-0.596	-0.583	-0.578	0.027	0.009	0.006	-0.584	-0.623	0.039	-0.630	0.000
4.824	-0.536	-0.526	-0.522	0.025	0.008	0.006	-0.528	-0.560	0.032	-0.566	0.000
Conf2	-3.186	-3.572	-3.642	0.169	0.059	0.043	-3.685	-3.355	-0.330	-12.556	-1.755
Conf3	-2.447	-3.388	-3.550	0.325	0.119	0.089	-3.639	-2.772	-0.867	-14.472	-2.215

r_{F---H}	$\Delta E_{el-prep}^{HF}$	$\Delta E_{el-prep}^{HF,X}$	$\Delta E_{el-prep}^{HF,Y}$	$E_{elstat} + \Delta E_{el-prep}^{HF}$	ΔE_{int}^{C-WP}	$\Delta E_{int}^{C-(T)}$	ΔE_{int}^{C-SP}	E_{res}^{C-SP}	E_{DISP}^{C-SP}
PES	on Conf1								
1.627	39.259	19.579	19.680	2.757	-0.105	-0.194	-0.910	0.552	-1.463
1.827	20.489	10.381	10.108	-0.735	-0.078	-0.131	-0.573	0.369	-0.941
2.026	10.314	5.294	5.020	-2.148	-0.054	-0.081	-0.320	0.343	-0.663
2.227	5.086	2.652	2.433	-2.520	-0.035	-0.044	-0.144	0.311	-0.455
2.427	2.472	1.305	1.167	-2.447	-0.027	-0.019	-0.028	0.313	-0.340
2.627	1.191	0.644	0.547	-2.214	-0.016	-0.005	0.029	0.272	-0.242
2.828	0.572	0.318	0.254	-1.945	-0.010	0.002	0.048	0.226	-0.178
3.028	0.278	0.161	0.117	-1.691	-0.007	0.007	0.066	0.196	-0.129
3.227	0.140	0.086	0.054	-1.468	-0.003	0.009	0.081	0.161	-0.080
3.430	0.077	0.050	0.027	-1.272	-0.002	0.010	0.064	0.118	-0.055
3.623	0.040	0.028	0.013	-1.131	-0.001	0.013	0.093	0.132	-0.039
3.823	0.026	0.018	0.007	-0.995	-0.003	0.012	0.082	0.111	-0.030
4.023	0.017	0.013	0.005	-0.879	-0.003	0.011	0.059	0.081	-0.022
4.223	0.012	0.009	0.003	-0.781	-0.002	0.010	0.046	0.064	-0.017
4.424	0.009	0.007	0.003	-0.696	-0.003	0.009	0.041	0.053	-0.012
4.624	0.007	0.005	0.002	-0.623	-0.003	0.008	0.034	0.043	-0.009
4.824	0.006	0.004	0.002	-0.560	-0.002	0.006	0.028	0.035	-0.007
Conf2	10.957	5.350	5.606	-1.600	-0.070	-0.053	-0.206	0.590	-0.796
Conf3	13.916	6.958	6.957	-0.557	-0.076	-0.132	-0.659	0.301	-0.960

4.4. BSSE-Corrected aug-cc-pVTZ Energies

$r_{\mathrm{F---H}}$	ΔE HF	ΔE CCSD	ΔE CCSD(T)	$\Delta E_{geo-prep}$ HF	$\Delta E_{geo-prep}$ CCSD	$\Delta E_{geo-prep}$ CCSD(T)	ΔE_{int}	ΔE_{int}^{HF}	ΔE_{int}^{C}	E_{elstat}	E_{exch}
PES	on	Conf1									
1.627	-2.176	-3.203	-3.432	0.654	0.188	0.132	-3.564	-2.830	-0.734	-36.285	-5.754
1.827	-3.443	-4.090	-4.240	0.346	0.079	0.046	-4.286	-3.790	-0.496	-21.063	-3.188
2.026	-3.457	-3.903	-4.004	0.289	0.063	0.036	-4.040	-3.746	-0.293	-12.398	-1.682
2.227	-3.097	-3.382	-3.446	0.234	0.049	0.026	-3.472	-3.331	-0.141	-7.594	-0.856
2.427	-2.660	-2.830	-2.867	0.186	0.037	0.019	-2.886	-2.846	-0.040	-4.924	-0.423
2.627	-2.256	-2.335	-2.356	0.148	0.028	0.013	-2.369	-2.404	0.034	-3.413	-0.203
2.828	-1.915	-1.931	-1.940	0.118	0.021	0.010	-1.950	-2.033	0.083	-2.527	-0.095
3.028	-1.637	-1.613	-1.614	0.094	0.016	0.007	-1.621	-1.731	0.110	-1.979	-0.043
3.227	-1.412	-1.364	-1.361	0.075	0.013	0.005	-1.366	-1.488	0.122	-1.616	-0.019
3.430	-1.215	-1.163	-1.159	0.067	0.011	0.004	-1.163	-1.283	0.120	-1.355	-0.009
3.623	-1.085	-1.006	-0.998	0.050	0.008	0.003	-1.001	-1.135	0.134	-1.175	-0.003
3.823	-0.955	-0.881	-0.873	0.042	0.007	0.002	-0.876	-0.997	0.121	-1.023	-0.001
4.023	-0.844	-0.777	-0.769	0.036	0.006	0.002	-0.771	-0.880	0.109	-0.899	-0.001
4.223	-0.750	-0.689	-0.682	0.032	0.005	0.002	-0.683	-0.782	0.098	-0.795	0.000
4.424	-0.669	-0.613	-0.607	0.028	0.004	0.002	-0.608	-0.697	0.089	-0.707	0.000
4.624	-0.599	-0.549	-0.542	0.025	0.004	0.001	-0.544	-0.624	0.081	-0.632	0.000
4.824	-0.539	-0.492	-0.485	0.022	0.003	0.001	-0.486	-0.562	0.075	-0.568	0.000
Conf2	-3.095	-3.268	-3.330	0.154	0.029	0.014	-3.344	-3.249	-0.094	-12.444	-1.754
Conf3	-2.439	-3.165	-3.304	0.297	0.063	0.034	-3.339	-2.736	-0.602	-14.468	-2.212

$r_{\mathrm{F---H}}$	$\Delta E_{el-prep}^{HF}$	$\Delta E_{el-prep}^{HF,X}$	$\Delta E_{el-prep}^{HF,Y}$	$E_{elstat} + \Delta E_{el-prep}^{HF}$	ΔE_{int}^{C-WP}	$\Delta E_{int}^{C-(T)}$	ΔE_{int}^{C-SP}	E_{res}^{C-SP}	E_{DISP}^{C-SP}
PES	on Conf1								
1.627	39.209	19.462	19.747	2.924	-0.058	-0.173	-0.504	0.847	-1.350
1.827	20.462	10.322	10.140	-0.601	-0.045	-0.117	-0.334	0.641	-0.975
2.026	10.334	5.296	5.037	-2.064	-0.032	-0.073	-0.188	0.469	-0.657
2.227	5.120	2.673	2.447	-2.474	-0.025	-0.041	-0.076	0.399	-0.475
2.427	2.501	1.322	1.179	-2.423	-0.020	-0.019	-0.001	0.327	-0.328
2.627	1.213	0.658	0.555	-2.201	-0.011	-0.006	0.052	0.270	-0.218
2.828	0.590	0.331	0.258	-1.938	-0.006	0.002	0.087	0.240	-0.154
3.028	0.291	0.170	0.121	-1.688	-0.004	0.008	0.105	0.208	-0.103
3.227	0.147	0.090	0.058	-1.469	-0.003	0.011	0.113	0.192	-0.079
3.430	0.080	0.051	0.029	-1.274	-0.002	0.011	0.111	0.169	-0.059
3.623	0.043	0.028	0.014	-1.132	-0.003	0.013	0.123	0.165	-0.042
3.823	0.028	0.019	0.009	-0.996	-0.002	0.012	0.111	0.142	-0.031
4.023	0.019	0.013	0.006	-0.880	-0.003	0.011	0.101	0.123	-0.023
4.223	0.014	0.010	0.004	-0.781	-0.003	0.011	0.091	0.107	-0.016
4.424	0.010	0.007	0.003	-0.697	-0.003	0.010	0.082	0.095	-0.012
4.624	0.008	0.006	0.002	-0.624	-0.002	0.009	0.074	0.084	-0.010
4.824	0.006	0.004	0.002	-0.562	-0.002	0.009	0.068	0.075	-0.007
Conf2	10.949	5.319	5.630	-1.496	-0.045	-0.047	-0.002	0.789	-0.791
Conf3	13.943	6.972	6.972	-0.525	-0.052	-0.110	-0.439	0.492	-0.931

4.5. BSSE-Corrected aug-cc-pVQZ Energies

r_{F---H}	ΔE HF	ΔE CCSD	ΔE CCSD(T)	$\Delta E_{geo-prep}$ HF	$\Delta E_{geo-prep}$ CCSD	$\Delta E_{geo-prep}$ CCSD(T)	ΔE_{int}	ΔE^{HF}_{int}	ΔE^{C}_{int}	E_{elstat}	E_{exch}
PES	on	Conf1									
1.627	-2.227	-3.426	-3.669	0.694	0.263	0.204	-3.873	-2.922	-0.951	-36.452	-5.753
1.827	-3.496	-4.252	-4.411	0.370	0.122	0.088	-4.499	-3.866	-0.633	-21.187	-3.189
2.026	-3.480	-3.981	-4.087	0.309	0.100	0.071	-4.158	-3.790	-0.368	-12.448	-1.682
2.227	-3.098	-3.420	-3.487	0.251	0.079	0.055	-3.542	-3.348	-0.194	-7.603	-0.856
2.427	-2.650	-2.842	-2.880	0.200	0.061	0.042	-2.922	-2.850	-0.072	-4.921	-0.423
2.627	-2.242	-2.337	-2.358	0.159	0.047	0.032	-2.390	-2.401	0.010	-3.407	-0.203
2.828	-1.900	-1.933	-1.943	0.126	0.037	0.025	-1.968	-2.027	0.058	-2.519	-0.095
3.028	-1.624	-1.614	-1.616	0.101	0.029	0.019	-1.636	-1.724	0.089	-1.971	-0.043
3.227	-1.401	-1.357	-1.354	0.081	0.023	0.015	-1.369	-1.482	0.113	-1.610	-0.019
3.430	-1.206	-1.165	-1.161	0.072	0.020	0.013	-1.175	-1.279	0.104	-1.351	-0.009
3.623	-1.079	-1.009	-1.001	0.053	0.015	0.010	-1.011	-1.132	0.121	-1.172	-0.003
3.823	-0.949	-0.872	-0.864	0.045	0.012	0.008	-0.872	-0.995	0.123	-1.021	-0.001
4.023	-0.840	-0.777	-0.769	0.039	0.011	0.007	-0.776	-0.878	0.102	-0.897	-0.001
4.223	-0.745	-0.691	-0.684	0.034	0.009	0.006	-0.690	-0.780	0.090	-0.794	0.000
4.424	-0.665	-0.615	-0.608	0.030	0.008	0.005	-0.614	-0.695	0.082	-0.706	0.000
4.624	-0.596	-0.550	-0.544	0.027	0.007	0.005	-0.549	-0.623	0.074	-0.631	0.000
4.824	-0.536	-0.493	-0.487	0.024	0.007	0.004	-0.492	-0.560	0.068	-0.566	0.000
Conf2	-3.143	-3.409	-3.475	0.165	0.049	0.034	-3.508	-3.308	-0.200	-12.530	-1.755
Conf3	-2.423	-3.270	-3.424	0.318	0.101	0.071	-3.495	-2.741	-0.754	-14.471	-2.214

r_{F---H}	$\Delta E^{HF}_{el-prep}$	$\Delta E^{HF,X}_{el-prep}$	$\Delta E^{HF,Y}_{el-prep}$	$E_{elstat} + \Delta E^{HF}_{el-prep}$	ΔE^{C-WP}_{int}	$\Delta E^{C-(T)}_{int}$	ΔE^{C-SP}_{int}	E^{C-SP}_{res}	E^{C-SP}_{DISP}
PES	on Conf1								
1.627	39.282	19.573	19.709	2.831	-0.085	-0.184	0.734	0.734	-1.416
1.827	20.510	10.385	10.125	-0.677	-0.064	-0.125	0.512	0.512	-0.955
2.026	10.340	5.309	5.031	-2.107	-0.045	-0.077	0.414	0.414	-0.661
2.227	5.111	2.668	2.443	-2.492	-0.031	-0.043	0.344	0.344	-0.463
2.427	2.494	1.318	1.175	-2.427	-0.024	-0.020	0.307	0.307	-0.335
2.627	1.209	0.656	0.553	-2.197	-0.014	-0.006	0.262	0.262	-0.232
2.828	0.588	0.329	0.259	-1.931	-0.009	0.002	0.233	0.233	-0.168
3.028	0.290	0.169	0.121	-1.681	-0.005	0.008	0.205	0.205	-0.118
3.227	0.148	0.090	0.058	-1.463	-0.003	0.011	0.185	0.185	-0.080
3.430	0.081	0.052	0.029	-1.270	-0.002	0.011	0.151	0.151	-0.056
3.623	0.043	0.029	0.014	-1.129	-0.002	0.013	0.151	0.151	-0.041
3.823	0.028	0.020	0.009	-0.993	-0.002	0.013	0.143	0.143	-0.030
4.023	0.020	0.014	0.006	-0.878	-0.003	0.012	0.116	0.116	-0.022
4.223	0.014	0.010	0.004	-0.779	-0.003	0.011	0.099	0.099	-0.017
4.424	0.011	0.008	0.003	-0.695	-0.003	0.010	0.087	0.087	-0.012
4.624	0.008	0.006	0.002	-0.623	-0.003	0.009	0.077	0.077	-0.010
4.824	0.006	0.005	0.002	-0.560	-0.002	0.008	0.069	0.069	-0.007
Conf2	10.977	5.356	5.620	-1.553	-0.060	-0.050	0.704	0.704	-0.794
Conf3	13.944	6.973	6.972	-0.527	-0.066	-0.124	0.385	0.385	-0.948

4.6. BSSE-Followed CBS Energies

r_{F---H}	ΔE HF	ΔE CCSD	ΔE CCSD(T)	$\Delta E_{geo-prep}$ HF	$\Delta E_{geo-prep}$ CCSD	$\Delta E_{geo-prep}$ CCSD(T)	ΔE_{int}	ΔE_{int}^{HF}	ΔE_{int}^{C}	E_{elstat}	E_{exch}
PES	on	Conf1									
1.627	-2.243	-3.564	-3.817	0.707	0.299	0.238	-4.055	-2.950	-1.105	-36.502	-5.752
1.827	-3.512	-4.346	-4.511	0.377	0.143	0.108	-4.619	-3.889	-0.730	-21.224	-3.189
2.026	-3.487	-4.028	-4.137	0.315	0.117	0.088	-4.225	-3.803	-0.422	-12.462	-1.682
2.227	-3.098	-3.447	-3.516	0.256	0.093	0.069	-3.585	-3.354	-0.232	-7.605	-0.856
2.427	-2.648	-2.854	-2.894	0.204	0.073	0.053	-2.947	-2.851	-0.096	-4.920	-0.423
2.627	-2.238	-2.344	-2.365	0.162	0.057	0.041	-2.407	-2.400	-0.007	-3.405	-0.203
2.828	-1.896	-1.941	-1.952	0.129	0.045	0.032	-1.984	-2.025	0.041	-2.517	-0.095
3.028	-1.619	-1.620	-1.623	0.103	0.035	0.025	-1.649	-1.722	0.074	-1.969	-0.043
3.227	-1.398	-1.356	-1.354	0.083	0.028	0.020	-1.374	-1.480	0.106	-1.609	-0.019
3.430	-1.203	-1.170	-1.167	0.074	0.025	0.018	-1.184	-1.277	0.093	-1.350	-0.009
3.623	-1.077	-1.014	-1.006	0.055	0.018	0.013	-1.019	-1.131	0.113	-1.171	-0.003
3.823	-0.948	-0.868	-0.859	0.046	0.015	0.011	-0.870	-0.994	0.124	-1.021	-0.001
4.023	-0.838	-0.779	-0.771	0.040	0.013	0.009	-0.781	-0.878	0.097	-0.897	-0.001
4.223	-0.744	-0.695	-0.688	0.035	0.011	0.008	-0.695	-0.779	0.084	-0.793	0.000
4.424	-0.664	-0.618	-0.611	0.031	0.010	0.007	-0.618	-0.695	0.076	-0.705	0.000
4.624	-0.595	-0.553	-0.546	0.027	0.009	0.006	-0.553	-0.622	0.069	-0.630	0.000
4.824	-0.535	-0.496	-0.490	0.025	0.008	0.006	-0.496	-0.559	0.063	-0.566	0.000
Conf2	-3.157	-3.489	-3.558	0.169	0.059	0.043	-3.601	-3.326	-0.275	-12.556	-1.755
Conf3	-2.418	-3.352	-3.516	0.325	0.119	0.089	-3.605	-2.743	-0.862	-14.472	-2.215

r_{F---H}	$\Delta E_{el-prep}^{HF}$	$\Delta E_{el-prep}^{HF,X}$	$\Delta E_{el-prep}^{HF,Y}$	$E_{elstat} + \Delta E_{el-prep}^{HF}$	ΔE_{int}^{C-WP}	$\Delta E_{int}^{C-(T)}$	ΔE_{int}^{C-SP}	E_{res}^{C-SP}	E_{DISP}^{C-SP}
PES	on Conf1								
1.627	39.305	19.607	19.698	2.803	-0.105	-0.192	-0.809	0.654	-1.463
1.827	20.524	10.404	10.120	-0.700	-0.078	-0.130	-0.522	0.420	-0.941
2.026	10.342	5.313	5.029	-2.120	-0.054	-0.080	-0.288	0.375	-0.663
2.227	5.108	2.666	2.442	-2.497	-0.035	-0.045	-0.151	0.304	-0.455
2.427	2.491	1.317	1.175	-2.428	-0.027	-0.020	-0.048	0.292	-0.340
2.627	1.208	0.655	0.553	-2.196	-0.016	-0.006	0.015	0.257	-0.242
2.828	0.588	0.329	0.259	-1.929	-0.010	0.002	0.049	0.228	-0.178
3.028	0.290	0.169	0.121	-1.679	-0.007	0.007	0.074	0.203	-0.129
3.227	0.148	0.090	0.058	-1.461	-0.003	0.010	0.099	0.180	-0.080
3.430	0.081	0.052	0.029	-1.269	-0.002	0.011	0.084	0.139	-0.055
3.623	0.043	0.029	0.014	-1.128	-0.001	0.013	0.101	0.140	-0.039
3.823	0.028	0.020	0.009	-0.993	-0.003	0.013	0.113	0.143	-0.030
4.023	0.020	0.014	0.006	-0.877	-0.003	0.012	0.089	0.111	-0.022
4.223	0.014	0.010	0.004	-0.779	-0.002	0.011	0.075	0.093	-0.017
4.424	0.011	0.008	0.003	-0.695	-0.003	0.010	0.070	0.082	-0.012
4.624	0.008	0.006	0.002	-0.622	-0.003	0.009	0.063	0.073	-0.009
4.824	0.006	0.005	0.002	-0.559	-0.002	0.008	0.058	0.065	-0.007
Conf2	10.985	5.368	5.618	-1.571	-0.070	-0.053	-0.153	0.643	-0.796
Conf3	13.944	6.973	6.972	-0.528	-0.076	-0.134	-0.652	0.308	-0.960

4.7. Plot of LED Terms

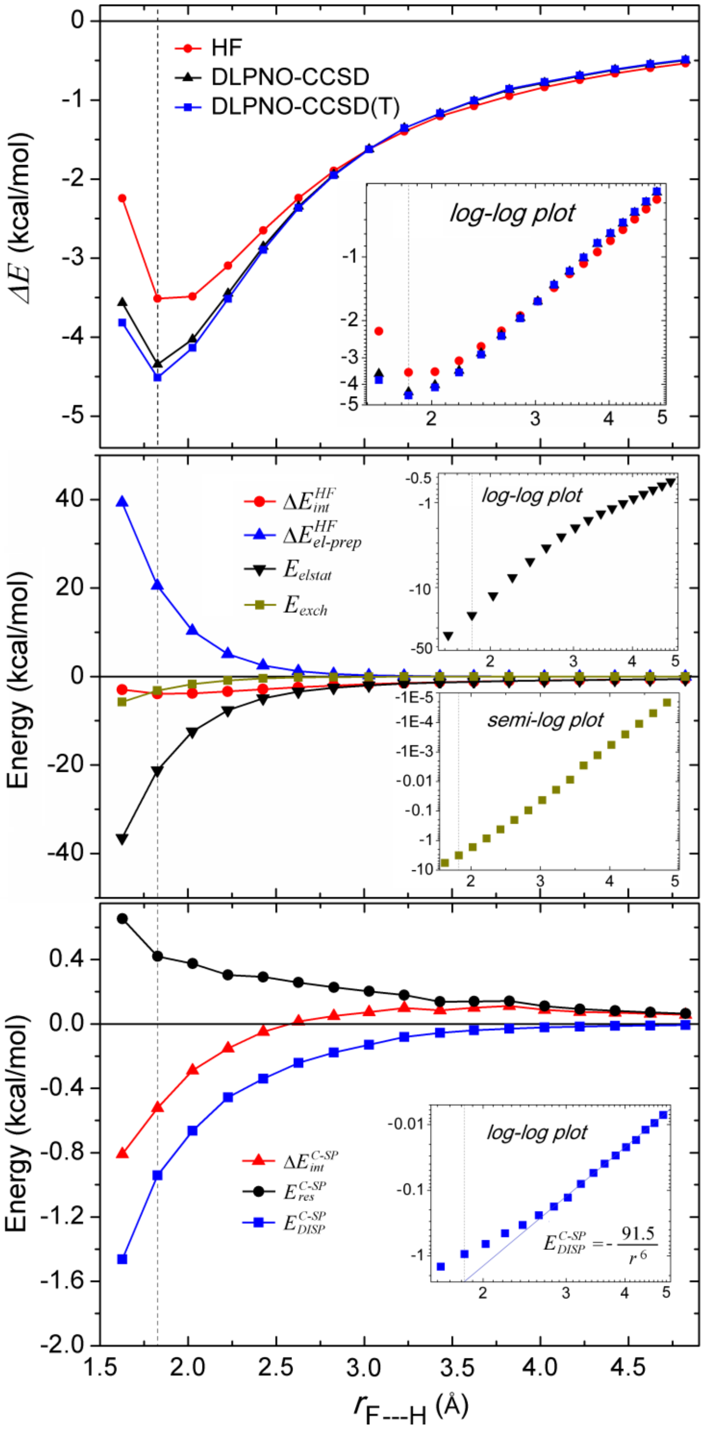

Figure S1: Dissociation curve (top), decomposed *HF* energy terms (middle), and significant decomposed correlation energy terms (bottom) of Conf1 of HF dimer as a function of the H-bond distance. The nearly linear relation of the long-range behavior of the dissociation energy, electrostatic and London dispersion energy terms in the log-log scale as well as the exchange energies in the semi-log scale are given as inserts on the graphs.

5. The Effect of Augmented Functions and Correction Scheme on ΔE_{int} (kcal/mol)

	cc-pVTZ	cc-pVQZ	CBS	aug-cc-pVTZ	aug-cc-pVQZ	CBS
W---W						
Without BSSE	-5.862	-5.379	-5.225	-5.120	-5.066	-5.029
With BSSE	-4.306	-4.662	-4.907	-4.707	-4.896	-5.014
HF---HF						
Without BSSE	-5.410	-4.883	-4.693	-4.784	-4.742	-4.706
With BSSE	-4.026	-4.323	-4.536	-4.286	-4.499	-4.619
